# An Engineered Soluble Single‐Chain TCR Engager for KRAS‐G12V Specific Tumor Immunotherapy

**DOI:** 10.1002/advs.202500181

**Published:** 2025-06-05

**Authors:** Keke Ma, Jie Wang, Min Jiang, Juanhua He, Fangyang Li, Dan Lu, Chao Su, Yan Chai, Wenjing Jin, Yu Chen, Catherine W.H. Zhang, Xiaopeng Ma, Hui Tan, George F. Gao, Shuguang Tan

**Affiliations:** ^1^ Department of Infectious Diseases Shenzhen Children's Hospital Shenzhen 518026 China; ^2^ CAS Key Laboratory of Pathogen Microbiology and Immunology Institute of Microbiology Chinese Academy of Sciences Beijing 100101 China; ^3^ Innovative Vaccine and Immunotherapy Research Center the Second Affiliated Hospital Zhejiang University School of Medicine Hangzhou 310009 China; ^4^ Medical School University of Chinese Academy of Sciences Beijing 100101 China; ^5^ YKimmu (Beijing) Biotechnology Co. Ltd Beijing 100000 China; ^6^ Department of Immunology Beijing Children's Hospital Capital Medical University National Centre for Children's Health Beijing 100045 China; ^7^ School of Medicine Zhongda Hospital Southeast University Nanjing 210009 China

**Keywords:** fine‐tuned TCR affinity, KRAS‐G12V mutation, TCR engagers, tumor inhibition

## Abstract

T cell receptor (TCR) based immunotherapy is an attractive strategy to target a wide range of intra‐tumoral antigens and elicit robust tumor cytotoxicity. However, engineering soluble TCR engagers that preserve physiological affinity is crucial for universal TCR drug development, yet remains challenging. In the present study, multiple TCR engagers featuring diverse architectures based on the KRAS‐G12V specific 1–2C TCR in the context of HLA‐A*11:01 is designed and evaluated. Notably, a soluble tandem double single‐chain TCR (STanD‐scTCR) engager, comprising two repeated single‐chain variable fragment (scFv) TCRs, exhibit enhanced binding avidity and potent T‐cell activation. Through site‐directed mutagenesis, T96F mutation (T96F‐TCR) within the TCR β chain is identified, which substantially augment T cell reactivity while maintaining physiological affinity and minimizing off‐target cross‐reactivity. The T96F‐mutated STanD‐scTCR engager demonstrates improved antigen sensitivity, promotes multi‐functional T‐cell responses, and facilitates immune synapse formation between T cells and target cells. In a xenograft tumor model harboring the KRAS‐G12V mutation, the TCR engager displays substantial tumor suppression efficacy. These findings underscore the therapeutic potential of 1–2C STanD‐scTCR engage in targeting KRAS‐G12V mutations in the context of HLA‐A*11:01. Furthermore, the engineering strategies employ in the development of STanD‐scTCR engager provide an invaluable for future designs of TCR engager drugs.

## Graphical abstract

This study develops a dimeric T cell receptor engager protein specific for tumor KRAS‐G12V mutation. The engager proteins are capable of mediating the formation of synapse between T cell and tumor cell and demonstrate substantial tumor suppression efficacy. The engineering strategies pave the way for the advancement of T cell receptor‐based tumor immunotherapy.

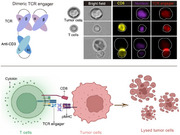

## 1 Introduction

T cell engagers (TCEs) have emerged as a promising strategy for treating solid tumors by recruiting and redirecting T cells to attack tumor cells through engagement of the CD3‐TCR complex and tumor antigen on target cells.^[^ ^1^ ^]^ TCEs typically incorporate an anti‐CD3 antibody as the CD3 binding arm and are broadly classified into two main strategies based on the tumor antigen‐binding arms, CD3 bispecifics and soluble TCR engagers.^[^ ^2^, ^3^ ^]^ CD3 bispecifics utilize antibodies to recognize surface antigens on tumor cells and have demonstrated promising therapeutic efficacy, as evidenced by the approval of IMDELLTRA (tarlatamab) in 2024 for small cell lung cancer and neuroendocrine prostate cancer.^[^ ^4^ ^]^ However, these therapies are constrained to surface‐expressed antigens, which represent only a subset of potential targets. On the other hand, soluble TCR engagers exploit the advantages of TCR‐directed targeting of intra‐ or extra‐cellular antigens presented by major histocompatibility complex (MHC, the MHC in humans is also known as human leukocyte antigen‐HLA) on the cell surface. Following the approval of KIMMTRAK (tebentafusp) in 2022 for metastatic uveal melanoma, soluble TCR engagers have been recognized as holding tremendous therapeutic potential for tumor immunotherapy.^[^ ^5^, ^6^, ^7^ ^]^

TCR binding to peptide/MHC (pMHC) complexes triggers immune synapse formation, involving co‐receptor engagement (e.g., CD8 for MHC class I) and TCR‐CD3 complex, which ultimately leads to T cell activation.^[^ ^8^, ^9^ ^]^ Natural TCRs typically exhibit physiological binding affinities ranging from 1 to 100 µM, whereas soluble TCR engagers reported usually employ high‐affinity TCRs with an affinity from pM to nM level.^[^ ^10^, ^11^, ^12^, ^13^ ^]^ The binding affinity of the TCR to pMHC ligand used in Kimmtrak is specifically engineered to reach as high as 80 pM.^[^ ^5^, ^14^ ^]^ However, increasing TCR affinity presents a double‐edged sword, as the improved binding affinity of maturated TCR comes with a cost in reduced specificity, antigen sensitivity and TCR‐mediated signal amplitude, even leading to lethal off‐target cytotoxicity.^[^ ^15^, ^16^, ^17^ ^]^ For instance, affinity‐enhanced TCR‐T cells targeting MAGE‐A3 cross‐recognize epitopes derived from MAGE‐A12 or TITIN proteins, resulting in patient mortality.^[^ ^15^, ^16^ ^]^ Recently, Andrew Poole et al. report an affinity‐enhanced TCR that recognizes KRAS‐G12D in the context of HLA‐A*11:01, but this TCR also exhibited substantial cross‐binding to the wildtype KRAS peptide.^[^ ^18^ ^]^ Furthermore, a TCR with excessively strong binding affinity may lead to early exhaustion of the T cells upon engagement with target cells, dampening the anti‐tumor immunity.^[^ ^19^, ^20^ ^]^ Therefore, engineering soluble TCR engagers with TCRs of physiological affinity holds substantial clinical significance for TCR‐based drug design. To enhance the effects of TCR‐based drugs, more research focused on improving TCR specificity by fine‐tuning TCR rather than TCR affinity maturation.^[^ ^17^, ^21^, ^22^ ^]^

Kirsten Rat Sarcoma Viral Oncogene Homolog (KRAS) mutations are critical drivers of tumor progression and metastasis, frequently occurring in colorectal cancer, pancreatic cancer, and lung cancer.^[^ ^23^ ^]^ Among the various KRAS mutations, those occurring at codons 12, specifically G12D, G12V and G12C, are the most prevalent across all tumor types.^[^ ^24^ ^]^ Consequently, KRAS‐G12 mutations are considered promising targets for tumor therapy. Small‐molecule drugs targeting the KRAS‐G12C have been approved for non‐small cell lung cancer, whereas drugs targeting the KRAS‐G12D mutant are currently under clinical investigation.^[^ ^25^, ^26^ ^]^ However, due to the lack of distinctive structural feature in the KRAS‐G12V protein, developing small molecule drugs specifically targeting KRAS‐G12V remains a challenge. The intracellular KRAS mutations generate neoantigens presented in the context of HLA‐A*11:01, ‐A*0301, ‐C*0802, etc.^[^ ^27^, ^28^ ^]^ Of note, KRAS mutations can also be presented by HLA class II alleles.^[^ ^29^ ^]^ Previously, our group and others have identified TCRs capable of specifically recognizing KRAS‐G12V peptides presented by HLA‐A11:01, inducing cytotoxic T cell responses against tumor cells with KRAS‐G12V mutations.^[^ ^30^, ^31^, ^32^ ^]^

Currently, the only reported TCR engager format is Immunocore's ImmTACs, comprises a soluble, full‐length extracellular TCR fused to an anti‐CD3 scFv.^[^ ^5^ ^]^ In the current study, utilizing the previously identified 1–2C TCR specific to KRAS‐G12V in the context of HLA‐A*11:01, we designed various soluble TCR engagers with distinct structural architectures and suggested the favorable TCR engager formats. Amongst these, a featuring tandem double single‐chain TCR engager (STanD‐scTCR engager) demonstrated robust T cell activation against the KRAS‐G12V antigen. Further fine‐tuning of the TCR through site‐directed mutagenesis, while maintaining physiological binding affinity, enhanced T cell reactivity and antigen sensitivity. In a xenogeneic tumor model, the site‐mutated TCR engager proteins exhibited remarkable tumor suppression efficacy. Collectively, we investigated the prospective TCR engager formats and enhanced the T cell activation by fine‐tuning TCR preserving physiological affinity. These findings provide an optimal soluble TCR design for the development of TCR engager drugs for solid tumor immunotherapy.

## 2 Results

### 2.1 Engineering of Soluble TCR Engagers with Varied Structural Architectures

The previously identified KRAS‐G12V specific 1–2C TCR was employed for engineering soluble TCR engagers, while a humanized anti‐CD3 UCHT1 antibody was chosen for fusion to re‐direct and activate T cells. The design of these soluble TCR engagers incorporating diverse structural architectures was driven by three pivotal considerations. First, to facilitate the construction of varied designs, both the TCR and anti‐CD3 antibody were formatted as scFv. Secondly, to enhance the TCR binding avidity, the TCR engagers were crafted with varied valences of TCRs, including monomeric, dimeric and tetrameric scFv TCRs per construct. Conversely, the number of anti‐CD3 antibodies was carefully restricted to one or two to mitigate potential cross‐linking of CD3 complexes, which may result in unexpected non‐specific T cell activation. Lastly, to minimize potential conformational hindrance that could impede the immune synapse formation, distinct constructions were devised with variable positioning of the TCR and anti‐CD3 antibody, either directly fused to each other or flanked by Fc domain.

**Figure 1:**
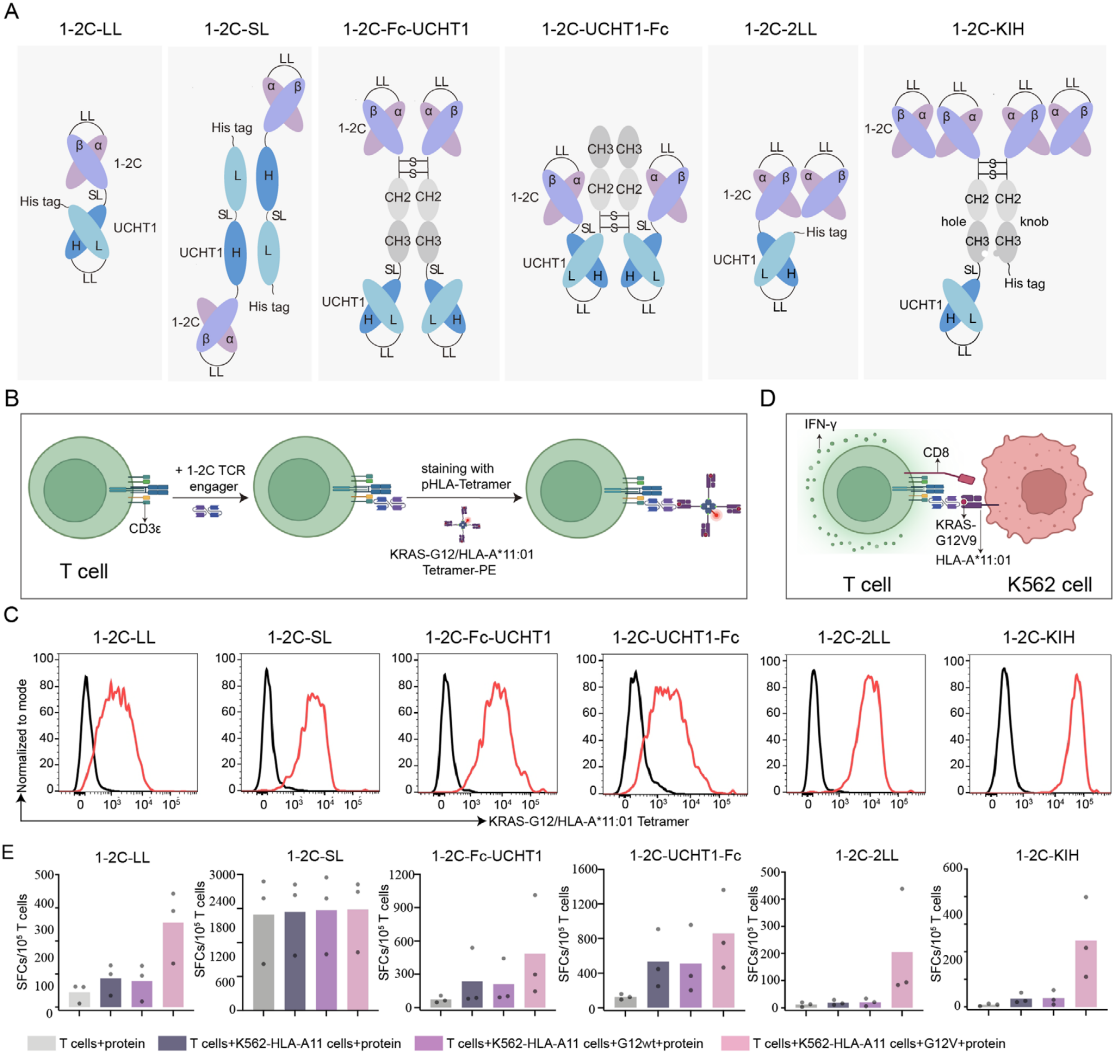
Specific binding and T cell responsiveness of varied 1–2C TCR engagers. A) Schematics representations of six soluble 1–2C TCR engagers architectures. LL, long linker; SL, short linker. UCHT1, anti‐CD3 scFv. The Vα and Vβ domains of 1–2C TCR are respectively labeled as α and β, whereas the VH and VL domains of UCHT1 antibody are labeled as H and L, accordingly. B) Schematic illustration of the binding assay of various 1–2C TCR engagers to T cells, as detected with the KRAS‐G12/HLA‐A*11:01 pHLA tetramer loaded with KRAS‐G12 wildtype (G12wt) or KRAS‐G12V peptides. Created with Biorender.com. C) Flow cytometry analysis of pHLA tetramers binding to T cells pre‐incubated with 1–2C TCR engager proteins. Tetramers were loaded with KRAS‐G12wt (black line) or KRAS‐G12V mutant peptides (red line). The data presented is representative of three independent experiments. D) Schematic depiction of the the functional evaluation assay, where 1–2C TCR engager s redirect T cells against the KRAS‐G12V antigen presented by K562‐HLA‐A11 cells, inducing IFN‐γ production. Created with Biorender.com. E) IFN‐γ ELIspot analysis of T cells co‐cultured with 1–2C TCR engager proteins (100 nM) and K562‐HLA‐A11 cells loaded with either KRAS‐G12wt or KRAS‐G12V peptides. Data points represent individual T cell responses from three donors and columns display the mean magnitude of IFN‐γ producing spots for the three donors.

To generate stable 1–2C scFv TCR, several mutations were introduced into the β chain, specifically G17E, H47Y, I77T and L80S, according to a prior study.^[^ ^33^, ^34^ ^]^ The 1–2C scFv was then fused to UCHT1 scFv using either long GS linker (LL, 3×G_4_S linker) or short GS linker (SL, 1×G_4_S linker). Six distinct soluble 1–2C TCR engagers were devised and designated (**Figure** **1**A). To eliminate Fc‐mediated effector function, the IgG4 Fc domain was employed with S228P/F234A/L235A mutations in the 1–2C soluble TCR engagers.^[^ ^35^ ^]^

These varied 1–2C TCR engager proteins were expressed with HEK‐293F cells and purified by affinity chromatography and gel‐filtration chromatography (Figure S1, Supporting Information). To evaluate the binding specificity of 1–2C TCR engagers, T cells were first co‐incubated with each of these TCR engager proteins separately. These TCR engagers successfully bound to CD3 molecules on T cells via the anti‐CD3 scFv (Figure 1B). Subsequently, the T cells attached with TCR engagers were detected with KRAS‐G12V/HLA‐A*11:01 peptide/HLA (pHLA) tetramer staining and analyzed by flow cytometry. The results demonstrated specific binding to KRAS‐G12V/HLA‐A*11:01 pHLA tetramer, whereas no binding was observed with the KRAS‐G12wt control tetramer. This indicates that the 1–2C TCR engagers preserved the binding capacity of both TCR and anti‐CD3 (Figure 1C).

To assess the potency of 1–2C TCR engagers in eliciting T cell activation, we examined IFN‐γ production by T cells co‐cultured with K562‐HLA‐A11 target cells loaded with KRAS‐G12V antigens (Figure 1D). The results revealed that 1–2C‐LL, 1–2C‐2LL and 1–2C‐KIH specifically induced T cell responses against the KRAS‐G12V peptide, whereas the other TCR engagers exhibited substantial non‐specific responses to the KRAS‐G12wt peptide (Figure 1E). Based on these findings, the 1–2C‐LL, 1–2C‐2LL and 1–2C‐KIH engagers were chosen for further investigation.

### 2.2 Multi‐Valent TCR Engagers Exhibit Enhanced Binding Avidity and T Cell Response

**Figure 2:**
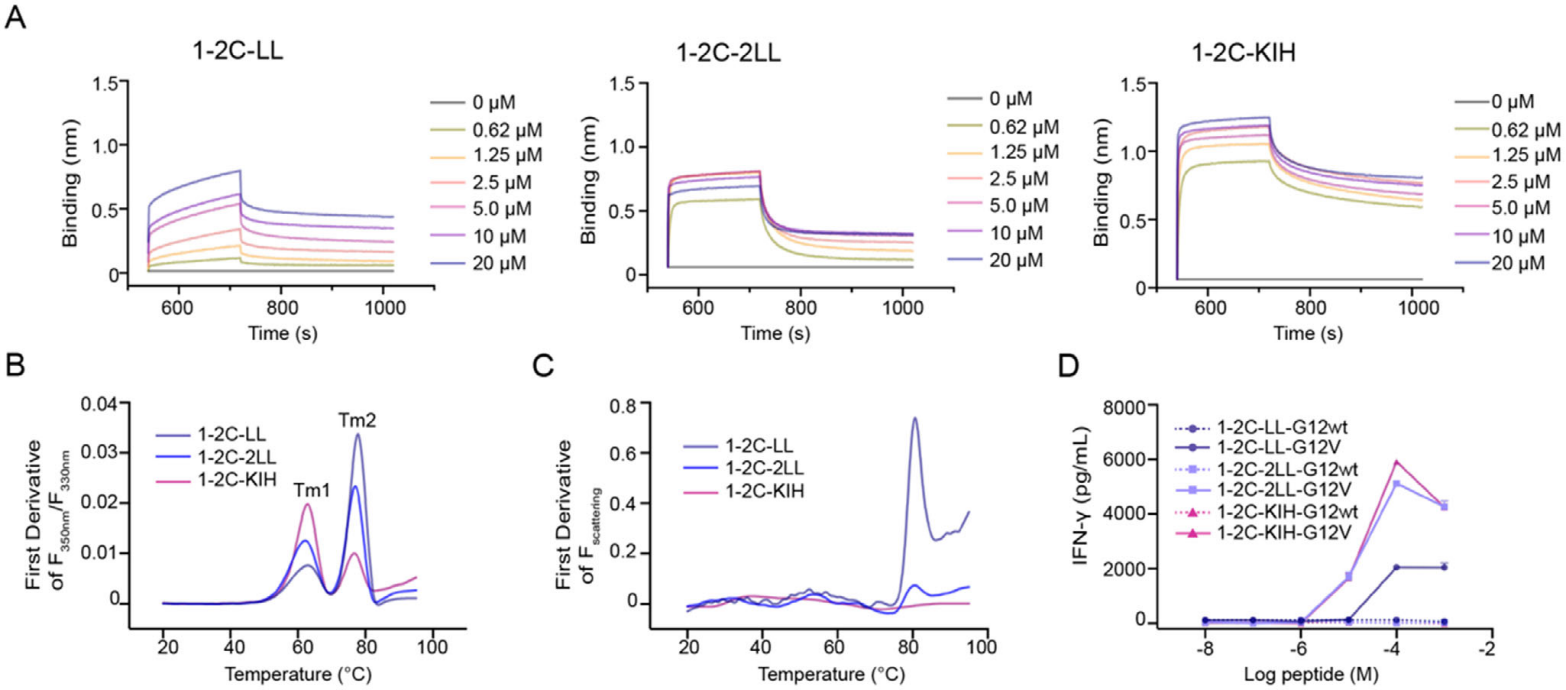
Binding characteristic, thermal stabilities and antigen sensitivity of 1–2C TCR engagers. A) Characterization of the binding profiles of 1–2C‐LL, 1–2C‐2LL, and 1–2C‐KIH TCR engagers, representing the mono‐, bi‐ and tetra‐valent TCRs, respectively, to the KRAS‐G12V/HLA‐A*11:01 pHLA ligand using BLI assay. The biotinylated KRAS‐G12V/HLA‐A*11:01 was immobilized on streptavidin‐coated probes, and serial dilutions of 1–2C engager proteins were flowed over the immobilized pHLA ligands. B) Thermal stability of 1–2C‐LL, 1–2C‐2LL, and 1–2C‐KIH TCR engagers assessed by NanoDSF, showing thermal transition temperatures. C) Aggregation propensity of 1–2C TCR engagers evaluated using NanoDSF by measuring their scattering temperatures. D) antigen sensitivity of 1–2C TCR engagers. T cells were co‐cultured with 1–2C TCR engager proteins (100 nM) and K562‐HLA‐A11 cells loaded with serial diluted KRAS‐G12wt or KRAS‐G12V peptides. The supernatants from the co‐cultures were analyzed using IFN‐γ ELISA to quantify the secreted IFN‐γ. Data points represent the mean concentration of IFN‐γ from duplicate wells, and the data are representative of three independent experiments.

We hypothesize that the design of multivalent TCRs can augment the binding avidity and enhance the T cell reactivity of the 1–2C TCR engagers. To substantiate this hypothesis, we examined both the binding capacity and functional potency of TCR engagers constructed with varying valences of TCR scFvs. The binding profiles of the KRAS‐G12V/HLA‐A*11:01 pHLA with different TCR engagers, 1–2C‐LL with monomeric TCR, 1–2C‐2LL with dimeric TCRs and 1–2C‐KIH with tetrameric TCRs, were assessed in a protein‐based Bio‐Layer Interferometry (BLI) binding assay. The results revealed that the dissociation rates of 1–2C‐2LL and 1–2C‐KIH TCR engagers were substantially slower than the monomeric 1–2C‐LL, and notably, the 1–2C KIH TCR engagers exhibited slowest dissociation rate. These finding indicate that increasing the TCR valence enhances the binding capability of 1–2C TCR engagers with KRAS‐G12V/HLA‐A*11:01 pHLA and extends TCR/pHLA interaction life‐time (**Figure** **2**A).

The conformational stability of the TCR engagers was evaluated using nano differential scanning fluorimetry (NanoDSF) through thermal denaturation. The analysis revealed two distinct unfolding peaks for the 1–2C‐LL, 1–2C‐2LL, and 1–2C‐KIH TCR engager proteins, corresponding to the thermal transition temperatures of the 1–2C scFv TCR and the UCHT1 scFv antibody (Figure 2B). Additionally, protein aggregation was monitored via scattering optics within the NanoDSF system. Notably, the 1–2C‐2LL and 1–2C‐KIH TCR engagers exhibited minimal aggregation during the experiments, whereas substantial aggregation was observed for the 1–2C‐LL at ≈80 °C (Figure 2C). Moreover, the 1–2C‐LL, 1–2C‐2LL, or 1–2C‐KIH TCR engager proteins were subjected to acidic, alkaline and oxidative conditions. Flow cytometry‐based binding assay demonstrated that the processed 1–2C‐2LL and 1–2C‐KIH TCR engagers maintained their binding capacity, whereas the binding capacity of the 1–2C‐LL decreased substantially (Figure S2, Supporting Information). These results indicate that the 1–2C‐2LL and 1–2C‐KIH TCR engagers exhibit robust stability under diverse extreme conditions.

The potency of T cell activation induced by the 1–2C‐LL, 1–2C‐2LL, and 1–2C‐KIH TCR engagers was evaluated through an IFN‐γ ELISA assay. The results revealed that the dimeric 1–2C‐2LL and tetrameric 1–2C‐KIH TCR engagers exhibited significantly enhanced antigen sensitivity and response strength compared to the monomeric 1–2C‐LL (Figure 2D). However, no substantial differences were observed between 1–2C‐2LL and 1–2C‐KIH TCR engagers. These findings indicate that increasing the valences of TCR scFv in the TCR engagers enhances T cell activity. Given the lack of improvement in antigen sensitivity or response magnitude with the 1–2C‐KIH architecture, the smaller‐sized 1–2C‐2LL engager was selected for further investigation.

### 2.3 Site‐Directed Fine‐Tuning Improves TCR Binding Capacity

**Figure 3:**
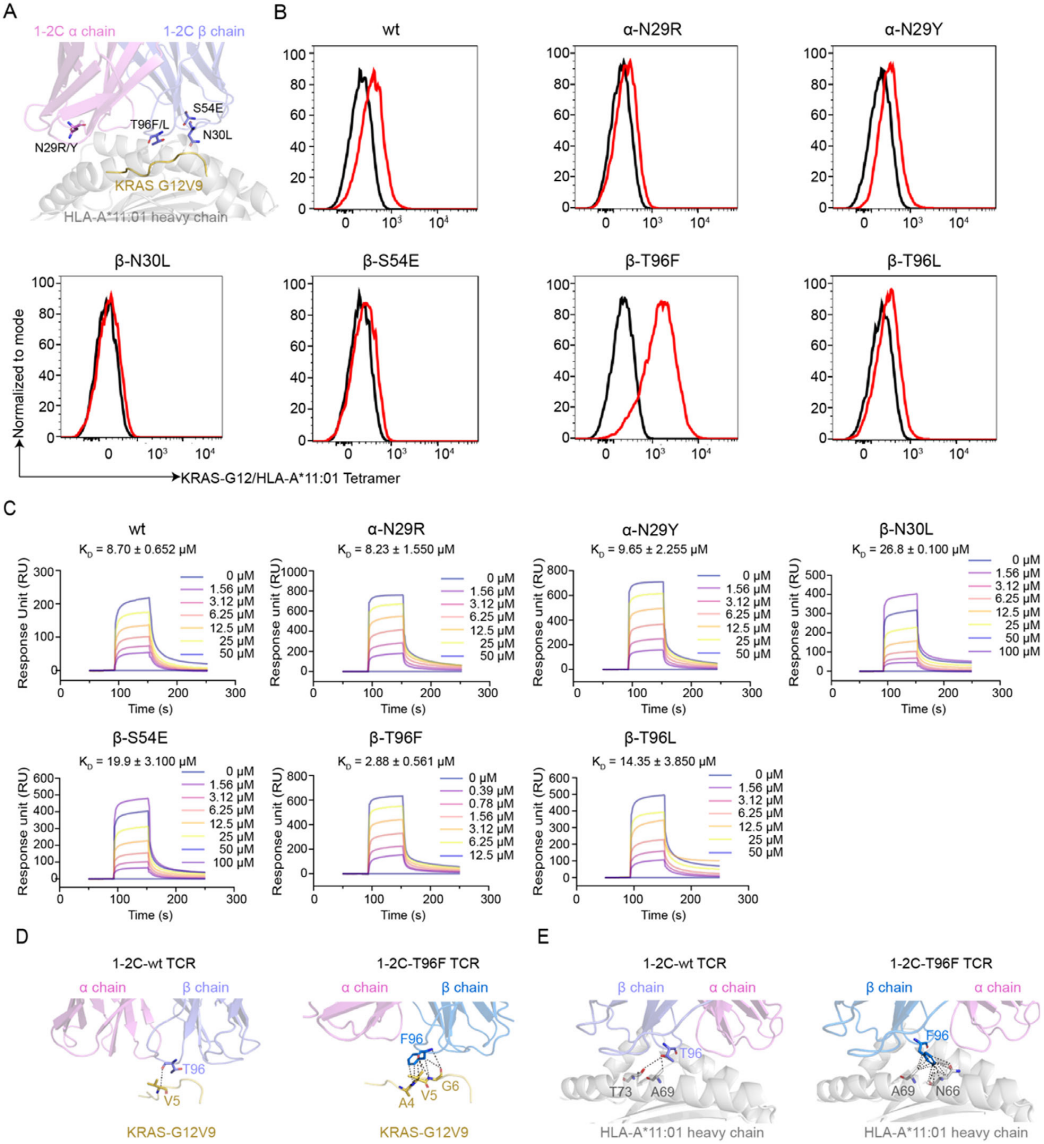
Site‐directed fine‐tuning of 1–2C TCR to enhance binding and T cell activating potency. A) Potential amino acid sites screened by Rosetta △G program are labeled in the 1–2C/KRAS‐G12V/HLA‐A*11:01 complex structure, with four key amino‐acid sites shown in stick. TCR/pMHC complex is shown in carton with 60% transparency. The 1–2C TCR is shown in voilet (α chain) and slate (β chain). The HLA‐A11 heavy chain is shown in gray90 and the peptide is shown in yellow. B) Flow cytometry analysis of pHLA tetramers loaded with KRAS‐G12wt (black line) or KRAS‐G12V mutant (red line) peptides binding to T cells incubated with varied site‐mutated 1–2C‐LL TCR engager proteins (50 nM). Wildtype 1–2C‐LL engager proteins were analyzed as control. The data shown represent one of three independent experiments. C) Kinetic analysis of 1–2C‐TCR mutants binding to pHLA. The biotinylated KRAS‐G12V/HLA‐A*11:01 was immobilized on streptavidin‐coated chips, and serial dilutions of varied mutant 1–2C‐LL engager proteins were flowed over the immobilized pHLA ligands. Data are represented as mean ± SEM, and results shown are representative of three independent experiments. D) Interaction network between T96 of the wildtype 1–2C TCR or F96 of the 1–2C‐T96F mutated TCR and KRAS‐G12V peptide. E) Interaction network between T96 of the wildtype 1–2C TCR or F96 of the 1–2C‐T96F mutated TCR and HLA‐A*11:01. The TCR, peptide and HLA‐A*11:01 are shown in carton with 60% transparency, residues involved in the interactions network are shown in stick, with dashed lines indicating van der Waals interactions. The 1–2C TCR is shown in violet (α chain) and slate (β chain). 1–2C‐T96F TCR is shown in violet (α chain) and marine (β chain). The HLA‐A*11:01 heavy chain is shown in gray90 and peptide is shown in yellow.

To optimize the binding capacity and T cell reactivity of 1–2C TCR, site‐directed mutagenesis was employed to fine‐tuning the interaction between 1–2C TCR and pHLA, while maintaining a physiological affinity range. The Rosette △G program was used to simulate amino acid conformations at all variable positions of 1–2C TCR in the TCR/pHLA interface, aiming to identify the residues and conformations that minimize the binding energy of the 1–2C/KRAS‐G12V/HLA‐A*11:01 complex. Single‐state design methodology was applied to determine amino acid substitutes. Potential mutations, focusing on complementary determined regions (CDRs), were calculated by modeling TCR interaction with either the KRAS‐G12V peptide or HLA‐A*11:01 (Figure S3A,B, Supporting Information). Finally, six favorable residue substitutes were chosen for their potential to strengthen the TCR/pHLA interaction, including N30L and T96F/L in the β chain, which contact with KRAS‐G12V peptide, and N29R/Y in the α chain and S54E in the β chain, which contact with HLA‐A*11:01 (**Figure** **3**A).

In a cell‐based flow cytometry binding assay, the results showed that the T96F mutation substantially enhanced the binding between 1–2C TCR and KRAS‐G12V/HLA‐A*11:01 pHLA, whereas the other mutations exhibited no substantial differences compared to the wild‐type 1–2C TCR (Figure 3B). To further investigated the binding characteristics of the 1–2C mutants, protein‐based Surface Plasmon Resonance (SPR) analyses were conducted. The results revealed that the T96F mutation displayed markedly improved binding affinity (K_D_ = 2.88 µM) compared to the wild‐type 1–2C TCR (K_D_ = 8.7 µM), yet remained within the physiological affinity range of natural TCRs (Figure 3C, Figure S3C,D, Supporting Information). In contrast, other mutants demonstrated either comparable (α‐N29R, α‐N29Y) or decreased (β‐N30L, β‐S54E, β‐T96L) binding affinities toward KRAS‐G12V/HLA‐A*11:01.

To further elucidate the mechanisms underlying the enhanced binding between TCR and pHLA caused by the T96F mutation, the structure of 1–2C‐T96F/KRAS‐G12V/HLA‐A*11:01 complex was determined using X‐ray crystallography at a resolution of 3.4 Å (Table S1 and Figure S4, Supporting Information). The electron density map at the TCR/pHLA interface is unambiguous (Figure S4C, Supporting Information). Analysis of the interaction network between F96 in CDR3β of 1–2C‐T96F TCR with both HLA‐A*11:01 and KRAS‐G12V peptide, we observed a significant increase in van der Waals interactions compared to the interaction network of T96 in wildtype 1–2C TCR (Figure 3D,E; Tables S2,S3, Supporting Information).

The potential cross‐reactivity of the T96F‐mutated 1–2C TCR was comprehensively evaluated. Flow cytometry‐based binding analyses demonstrated that no detectable binding to wildtype KRAS or other KRAS mutant pHLA tetramers (Figure S3E, Supporting Information). Given the focus on the 1–2C‐2LL TCR engager, a combinatorial peptide library screening was performed to further investigate whether T96F‐mutated 1–2C‐2LL TCR engager (also referred as 1–2C‐2LL‐T96F TCR engager) proteins could induce cross‐reactivity with homologous peptides in the human genome. This library comprised peptides in which each residue of the KRAS‐G12V peptide was substituted with 20 amino acids (Figure S5A, Supporting Information). T cells responses from two donors induced by T96F‐mutated 1–2C‐2LL TCR engager proteins were analyzed using IFN‐γ ELISA (Figure S5B–J, Supporting Information). ScanProsite (https://prosite.expasy.org/scanprosite/) was employed to screen for homologous peptides in the human genome that matched the responsive motifs observed in the combinatorial peptide library analysis. No homologous peptides were identified. We further analyzed the cross‐reactivity to homologous peptides exist in the human genome that show sequence similarity with KRAS‐G12V peptide (Figure S6A, Supporting Information). Through BLAST analyses, 16 homologous peptides were selected from 14 proteins for cross‐reactivity analyses (Table S4, Supporting Information). No cross‐reactivity was observed for any of the homologous peptides. Potential off‐target effects were further investigated against varied cell lines expressing different HLA types, and no substantial responses could be observed upon co‐culturing with any of these varied cell lines (Figure S6B,C, Supporting Information). These findings suggest that the T96F‐mutated 1–2C‐2LL TCR engager exhibits high specificity for KRAS‐G12V and a low likelihood of off‐target toxicity, indicating its safety profile and limited risk of cross‐reactivity with self‐antigens.

### 2.4 T96F‐Mutated 1–2C‐2LL Engager Substantially Enhance T Cell Activity

**Figure 4:**
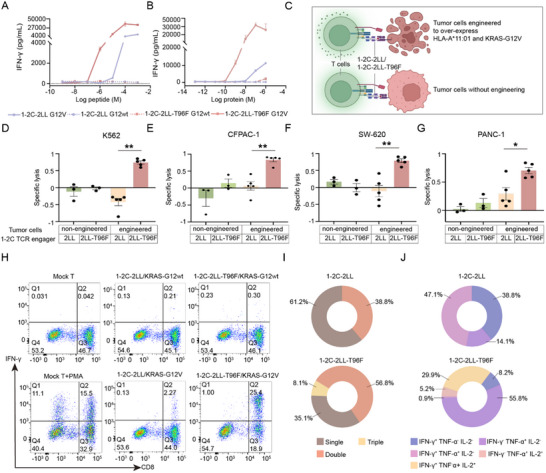
T96F‐mutated 1–2C‐2LL TCR engager potentially enhanced T cell reactivity and tumor cytotoxicity. A) T cells were co‐cultured with 1–2C‐2LL TCR engager proteins (100 nM) and K562‐HLA‐A11 cells loaded with serially diluted KRAS‐G12wt or KRAS‐G12V peptides. Secreted IFN‐γ in the supernatants was quantified using ELISA. B) T cells were co‐cultured with serially diluted 1–2C‐2LL TCR engager proteins and K562‐HLA‐A11 cells loaded with KRAS‐G12wt or KRAS‐G12V peptides (10 µg mL^−1^). Secreted IFN‐γ in the supernatants was quantified by ELISA. Data points represent the mean concentration of IFN‐γ from triplicate wells in (A) and (B). The data are representative of three independent experiments. C) Schematic of the flow cytometry‐based cytotoxicity assay, where 1–2C‐2LL TCR engager re‐direct T cell to target tumor cells engineered over‐expressing KRAS‐G12V and HLA‐A*11:01. Tumor cells without engineering served as control. created with BioRender.com. D–G) Cytotoxicity analysis of T cells against K562 cells D), CFPAC‐1 cells E), SW‐620 cells F), and PANC‐1 cells G) engineered to over‐express KRAS‐G12V/HLA‐A*11:01. T cells were co‐cultured with tumor cells and 1–2C‐2LL TCR engager proteins (100 nM). The T cells cytotoxicity was assessed by flow cytometry and the specific lysis of tumor cells was calculated as [(tumor cell events in PBS treatment − tumor cell events in TCR engager treatment)/tumor cell events in PBS treatment]. The data points represent T cell responses from at least three donors, with mean ± SEM values shown as columns. Statistical significance was calculated with an unpaired Student's t test (**p < 0.01, *p < 0.05). H) IFN‐γ Cytokine‐producing profiles induced by 1–2C‐2LL TCR engagers. T cells were co‐cultured with wildtype or T96F‐mutated 1–2C‐2LL engager proteins in the presence of K562‐HLA‐A11 cells loaded with KRAS‐G12wt or KRAS‐G12V peptides, and intracellular cytokine staining (ICS) was performed to detect the secretion of IFN‐γ. The number in the panel represents the percentage of cytokine‐staining positive cells. I and J) Functional profiling of KRAS‐G12V specific CD8^+^ T cells induced by wild‐type or T96F‐mutated 1–2C‐2LL engagers. Percentages of mono‐functional (single) T cells producing one cytokine (IFN‐γ, TNF‐α, or IL‐2), dual‐functional (double) T cells producing two cytokines, and multi‐functional (triple) T cells producing all three cytokines were determined. The data shown are representative of two independent experiments.

To examine whether the T96F mutation enhances the capacity of 1–2C‐2LL TCR engagers in inducing specific T cell reactivity against KRAS‐G12V antigen, a comprehensive investigation of T cell responses was performed. Antigen sensitivity was evaluated with serial diluted KRAS‐G12V peptides co‐cultured with a fixed concentrations of 1–2C TCR engager proteins. Conversely, sensitivity to the TCR engagers was assessed with serial dilutions of TCR engager proteins co‐cultured with fixed concentrations of antigens. The results demonstrated that the T96F‐mutated 1–2C‐2LL engager elicited significantly increased antigen sensitivity and TCR engager sensitivity for specific induction of IFN‐γ production, as compared to wildtype 1–2C‐2LL TCR engager (**Figure** **4**A,B). Flow cytometry‐based cytotoxicity assay further demonstrated that the T96F‐mutated 1–2C‐2LL engager exhibited specific immune response and substantial cytotoxicity against various target cells, including K562 cell, SW‐620 cell, CFPAC‐1 cell and PANC‐1 cells, which were genetically engineered to over‐express HLA‐A*11:01 and KRAS‐G12V (Figure 4C–G; Figures S7,S8, Supporting Information). In contrast, minimal or barely perceptible cytotoxicity was observed with the wild‐type 1–2C‐2LL engager proteins.

Previous studies have demonstrated that CD4^+^ and/or CD8^+^ T cells capable of producing multiple cytokines, known as polyfunctional T cells, may exhibit enhanced functional competence.^[^ ^36^ ^]^ Therefore, we analyzed the proportion of single‐cytokine‐secreting and multiple‐cytokine‐secreting T cells elicited by 1–2C‐2LL TCR engagers using an intracellular cytokine staining (ICS) assay. The results revealed that the T96F‐mutant 1–2C‐2LL TCR engager substantially induced higher frequencies of IFN‐γ and TNF‐α‐producing T cells compared to wildtype 1–2C‐2LL (Figure 4H; Figure S9A, Supporting Information). Notably, the T96F‐mutated 1–2C‐2LL TCR engager also induced IL‐2‐producing T cells, whereas no IL‐2 production was observed with the wildtype 1–2C‐2LL (Figure S9B, Supporting Information). All cytokine‐producing T cells were identified as CD8^+^ T cells, highlighting the CD8‐dependency of T cell responses induced by 1–2C‐2LL TCR engagers.

A detailed examination of the functional profiles of cytokine‐producing T cell subsets induced by 1–2C‐2LL engager proteins revealed that both T96F‐mutated and wildtype 1–2C‐2LL TCR engagers elicited monofunctional or polyfunctional T cell responses, producing either one or two+ cytokines, respectively. Notably, the T96F‐mutated 1–2C‐2LL TCR engager induced a significantly higher proportion of polyfunctional T cells capable of producing more than two cytokines compared with wildtype 1–2C‐2LL. The T cell subset producing all three cytokines (IFN‐γ^+^ TNF‐α^+^ IL‐2^+^) was exclusively observed in response to the T96F‐mutated 1–2C‐2LL TCR engager (Figure 4I,J). These results suggest that the T96F‐mutated 1–2C‐2LL TCR engager substantially increase the frequencies of cytokine‐producing T cells and induce a more robust polyfunctional T cell response.

### 2.5 T96F‐Mutated 1–2C‐2LL Engager Facilitate the Synapse Formation and Mediate Attachment between T Cells and Target Cells

**Figure 5:**
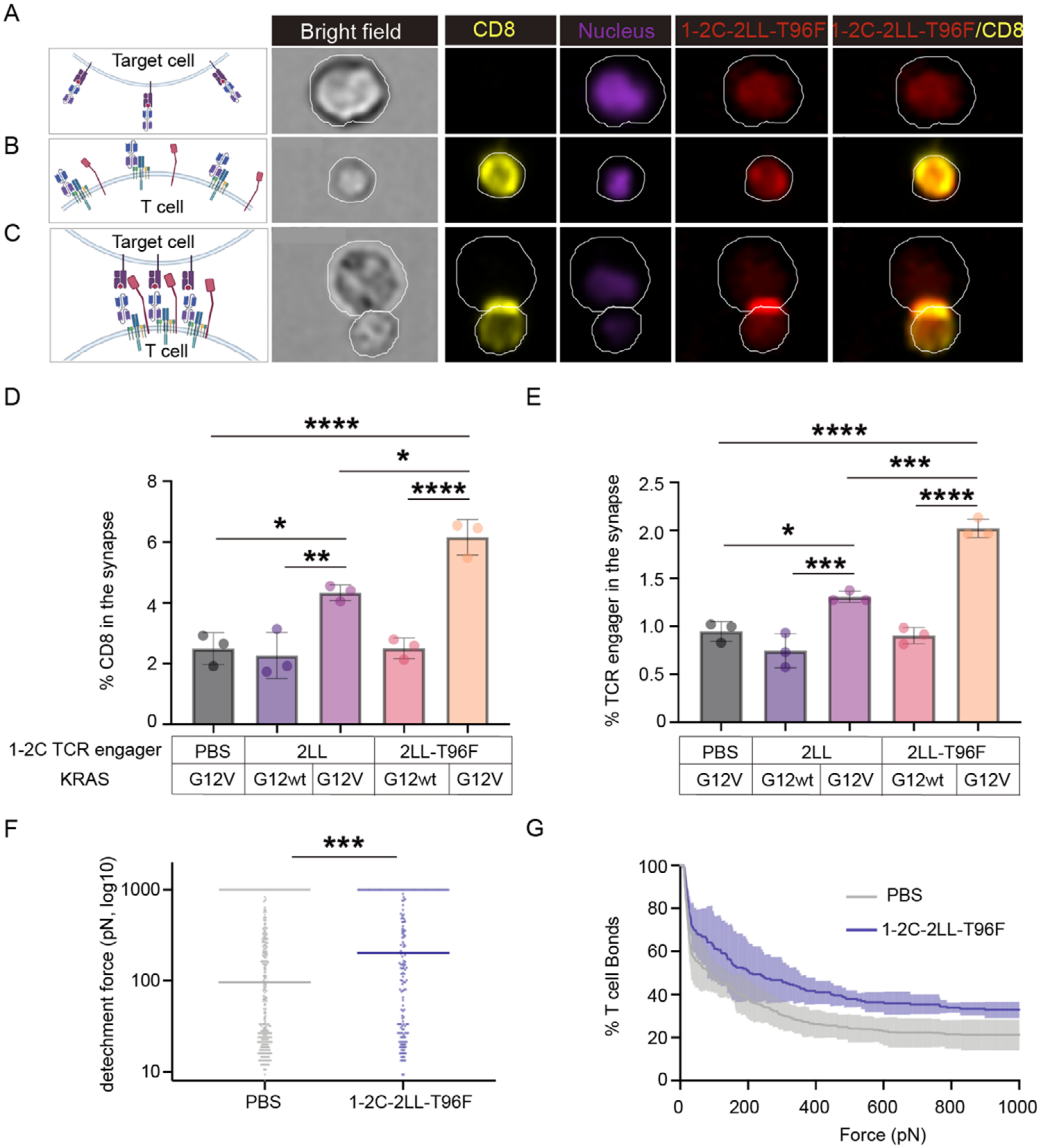
1–2C‐2LL TCR engagers facilitated immune synapses formation between T cells and target cells. A–C) Representative immunofluorescence images depicting the co‐cultures of T96F‐mutated 1–2C‐2LL TCR engager proteins with A) K562‐HLA‐A11 target cells loaded with KRAS‐G12V peptide, B) T cells, or C) both T cells and K562‐HLA‐A11 target cells loaded with KRAS‐G12V peptide. The co‐cultured cells were stained with fluorescence‐labeled anti‐CD8 antibodies and fluorescence‐labeled anti‐his‐tag antibodies, with cell outlines indicated by white lines. The data are representative of two independent experiments. D and E) Quantification of the percentage of CD8 molecules D) and 1–2C‐2LL TCR engagers E) accumulation at the immunological synapse. Data are represented as means ± SEM, and statistical significance was calculated using ordinary one‐way ANOVA test with Kruskal‐Wallis multi‐comparisons. (****p < 0.0001, ***p < 0.001, **p < 0.01, *p < 0.05). Data are representative of two independent experiments. F and G) Cell avidity measurements using z‐Movi technology evaluate T cells binding to SW‐620‐HLA‐A11‐luci target cells in the presence of T96F‐mutated 1–2C‐2LL engager proteins. F) The median force required to detach T cells from SW‐620‐HLA‐A11‐luci cells, with each point representing an individual T cell in the microfluidic chips. Differences were analyzed using an unpaired Student's t test (***: *p* < 0.001). G) The binding frequency of T cells to SW‐620‐HLA‐A11‐luci cells as the applied force increases. T cells and target cells co‐cultured with PBS were enrolled as negative control. The data presented are representative of two independent experiments.

Efficient T cell activation relies on the clustering of the TCR‐CD3 complex at the immune synapse, a specialized interface facilitating antigen recognition and downstream signaling.^[^ ^37^, ^38^ ^]^ To evaluate whether the 1–2C‐2LL TCR engagers could promote immune synapse formation between T cells and target cells, immunofluorescence imaging assay were performed using T cells incubated with 1–2C‐2LL engager proteins and K562‐HLA‐A11 cells loaded with KRAS‐G12V peptide, while K562‐HLA‐A11 cells loaded with KRAS‐G12wt peptides were served as control. The results demonstrated that the TCR engager proteins clustered at the artificial synapse between T cells and target cells, indicating that 1–2C‐2LL TCR engagers effectively induce TCR/CD3 complex clustering, mimicking a natural immune synapse. Additionally, CD8 molecules clustering were observed at the synapse, suggesting that synapse formation induced by the TCR engagers depend on CD8 co‐receptor aggregation (**Figure** **5**A–C). Quantitative analysis of fluorescence intensity within and outside the immune synapse revealed that the T96F‐mutated 1–2C‐2LL TCR engager significantly enhanced the clustering of CD8 molecules and TCR engagers compared with the wild‐type 1–2C‐2LL, highlighting its potential to enhance T cell activation (Figure 5D,E).

To further identify whether the T96F‐mutated 1–2C‐2LL engagers could mediate attachment between T cells and tumor cells, cell‐based avidity testing was conducted using z‐Movi technology with SW620‐HLA‐A11‐luci target cells pre‐coated on microfluidic chips. The chips were then incubated with T cells and the T96F‐mutated 1–2C‐2LL engager proteins simultaneously.^[^ ^39^ ^]^ In this system, an acoustic force ranging from 0 to 1000 pN was gradually applied, with T cells detachment from target cells monitored as the force increased. The results demonstrated that a substantial proportion of T cells remained bound to the target cells in the presence of the T96F‐mutated 1–2C‐2LL engager proteins, even under increasing acoustic force (Figure 5F,G). This finding indicates that the T96F‐mutated 1–2C‐2LL TCR engager mediated substantial cell‐cell attachment between T cells and target cells.

### 2.6 T96F Mutated 1–2C‐2LL TCR Engager Displays Tumor Suppression Efficacy in a Xenograft Model

**Figure 6:**
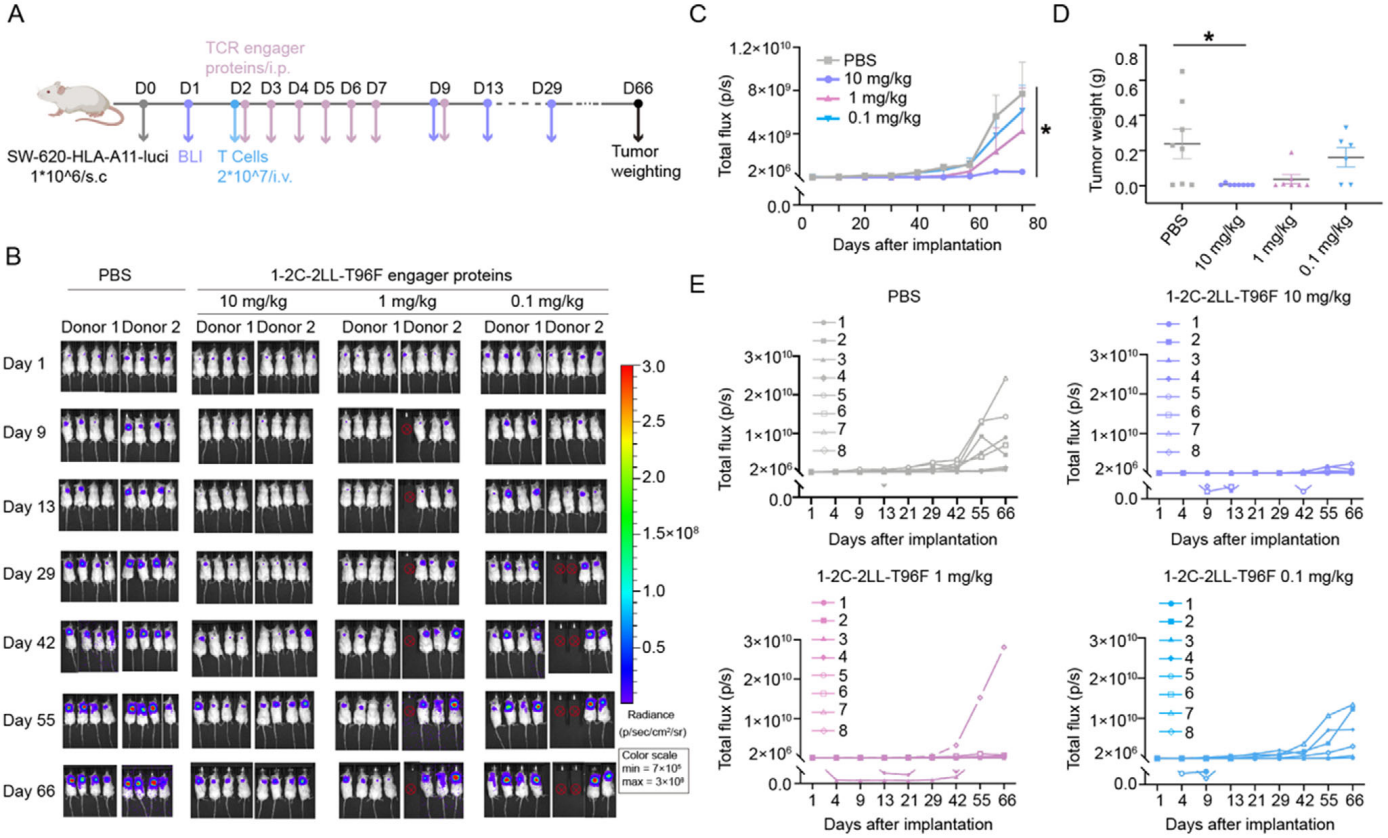
Tumor inhibition efficacy of T96F‐mutated 1–2C‐2LL engage in a tumor bearing mouse model. A) Schematic representation of the experimental process for the colorectal cancer mouse model, created with BioRender.com. NCG mice were inoculated subcutaneously with SW‐620 cells stably expressing HLA‐A11 and a luciferase reporter (1×10^6) (SW‐620‐HLA‐A11‐Luci) on day 0 (D0). On day 2 (D2), T cells derived two donors were intravenously injected. Three doses (10, 1, 0.1 mg kg^−1^) of the T96F‐mutated 1–2C‐2LL TCR engager were administered via intraperitoneal injection. Tumor burdens were tracked using bioluminescence imaging (BLI) for 66 days, and tumor weights were measured after sacrificing the mice at the end of the experiment. B) Bioluminescence images of mice from the indicated treatment groups over 66 days. The red × symbols within circle denote deceased mice. C) Statistics analysis of tumor fluorescence values for each treatment group, with data point representing the mean ± SEM. Statistical significance was calculated using Ordinary one‐way ANOVA test with Kruskal‐Wallis multi‐comparisons for each group. (*p < 0.05). D) The tumor weights of each group from sacrificed mice at the end of the experiment are presented, with n = 8 mice per group. Statistical significance was calculated with an Ordinary one‐way ANOVA test with Kruskal‐Wallis multi‐comparisons (*p < 0.05). E–H) Longitudinal tracking of tumor fluorescence values for individual mice in each treatment group, with each line representing changes in fluorescence values for a single mouse.

To investigate the in vivo anti‐tumor efficacy of the T96F‐mutated 1–2C‐2LL TCR engager, SW‐620‐HLA‐A11 cells stably expressing luciferase reporter (SW‐620‐HLA‐A11‐Luci) were subcutaneously (s.c.) implanted into NOD‐*Prkdc* ^em26Cd52^ *Il2rg* ^em26Cd22^/NjuCrl (NCG) mice to establish a colorectal cancer model (**Figure** **6**A).^[^ ^30^ ^]^ T cells from two donors were injected intravenously (i.v.) and three varied doses of TCR engager protein group (i.e., high, medium, and low doses with 10, 1, and 0.1 mg kg^−1^, respectively) were infused intraperitoneally (i.p.). The intraperitoneal administration route was specifically selected based on pharmacokinetic profiling demonstrating superior drug persistence compared to intravenous delivery (Figure S10, Supporting Information). PBS was administrated as negative control in parallel. Tumor burden was monitored over time using bioluminescence imaging (BLI) (Figure 6B).

The results showed that tumor growth was significantly inhibited in high‐dose group compared to the PBS group, whereas no significant difference were observed in the medium‐ and low‐dose groups relative to the PBS treatment group (*p* < 0.05) (Figure 6C). An individual fluorescence tracking revealed that, with the exception of one mouse in the medium‐dose group that had an unusually high tumor load, the tumors in this group were generally lower than those in the PBS treatment group (Figure 6E). At 66 days post implantation, the mice were euthanized, and the tumor tissues were excised and weighed (Figure S11, Supporting Information). Consistently, the tumor weights in the high‐dose engager protein group were significantly lower than those in the PBS treatment group (*p* < 0.05), while the medium‐ and low‐dose groups did not exhibit significant differences compared to the PBS treatment group (Figure 6D). These findings indicate that the T96F‐mutated 1–2C‐2LL engager is capable of inducing substantial in vivo anti‐tumor efficacy.

## 3 Discussion

Unlike the CD3‐bispecifics therapeutics that have been approved for clinical use or a under clinical investigation, the development of universal TCR engager drugs remains challenging due to the intrinsic nature of response for B/T cells.^[^ ^11^, ^40^, ^41^ ^]^ The structural architectures of CD3‐bispecifics are extensively varied, e.g. DVD‐Ig, IgG‐scFv, crossMab, BiTE.^[^ ^42^ ^]^ However, the structural designs of soluble TCR engager drugs are considerably constrained.^[^ ^43^ ^]^ One substantial challenge in soluble TCR engineering is the instability of the variable domains of TCR α and β chains when deprived of constant domains, whereas the variable domains of antibodies are typically stable. In the case of KIMMTRAK the extracellular domain of TCR, encompassing both variable and constant domains of the α and β chain, was fused to an anti‐CD3 scFv antibody.^[^ ^5^ ^]^ Here, we engineered scFv from 1–2C TCR, incorporating mutations in the Vβ domain, which conferred substantially stability under a variety of extreme conditions.

Previous studies suggest that the optimal intercellular distance between a T cell and a target cell for effective is ≈15 nm, equivalent to the span of four Ig‐like domains.^[^ ^44^, ^45^ ^]^ This distance corresponds to the vertical separation of the natural TCR/pMHC complex, where the extracellular region of pMHC, composed of two Ig‐like domains, interacts with the TCR, which also possesses a two Ig‐like domain extracellular region. Besides, the engagement of CD4/CD8 co‐receptors is thought to be crucial for initiating the intracellular signaling by stabilizing the TCR/pMHC complex.^[^ ^37^, ^46^ ^]^ Consequently, the structural architecture of soluble TCR engagers plays a pivotal role in modulating the TCR synapse formation, including CD8 coreceptors engagement, ultimately influencing the strength and specificity of the induced T cell response.

Our previous investigations have established that the IgG‐scFv format bispecific antibody manifests a synergistic neutralizing effect against SARS‐COV‐2.^[^ ^47^, ^48^ ^]^ We engineered three TCR engager formats with Fc, 1–2C‐Fc‐UCHT1, 1–2C‐UCHT1‐Fc, and 1–2C‐KIH. Both 1–2C‐Fc‐UCHT1 and 1–2C‐UCHT1‐Fc activate T cells non‐specifically, likely due to the CD3 cross‐linking mediated by the bivalent CD3 antibody, a mechanism resembling that 1–2C‐SL. Distinct from anti‐CD3 antibodies, which demand the utmost monovalency, an augmentation in TCR valence confers an advantage upon the TCR engager. Notably, the dimeric 1–2C‐2LL and tetrameric 1–2C‐KIH TCR engagers demonstrated superior antigen binding capacity, antigen sensitivity, and stability in comparison to the monomeric 1–2C‐LL.

Comparative analyses revealed that the 1–2C‐2LL TCR engager could induce specific T cell responses compared with the other constructs, with small‐scale and substantial stability. To characterize the architecture of 1–2C‐2LL structure, we re‐named the 1–2C‐2LL engager as 1–2C STanD‐scTCR engager. The 1–2C STanD‐scTCR engager preserved the minimum domains required for pMHC engagement and achieved T cell activation through the anti‐CD3 scFv antibody. The ICS analyses of the 1–2C STanD‐scTCRs engager in stimulating cytokine expression demonstrated that the activation was CD8‐dependent, indicating its retention of CD8 co‐receptor engagement potency. Therefore, the engineered STanD‐scTCR engager represents an optimal architecture for the engagement to pMHC and induction of T cell activation. The 1–2C‐KIH asymmetry architecture also exhibited substantial specificity and folding stability, albeit posing challenges for large‐scale production of these asymmetry recombinant proteins in industry. The integration of the Fc fragment into the 1–2C‐KIH asymmetry architecture holds the promise of enhanced in vivo durability. Furthermore, the 1–2C KIH offer potential for further engineering by incorporating cytokines, coreceptors, or chemokines to improve anti‐tumor T cell function or tumor infiltration. Collectively, the TCR engager architectures developed in this study provide promising framework for designing universal TCR engager drugs.

The evolutionary advantage of TCR is scanning “non‐self” target cells with lower affinity compared with antibody. Notably, the epitope density required for T cell activation is significantly lower for TCR‐T cells than for antibody‐based CAR‐T cells, with 1 to 50 versus 10^3^ epitopes per cell, respectively.^[^ ^40^ ^]^ However, excessive affinity enhancement of TCR for pMHC leads to non‐specific recognition of self‐derived peptides.^[^ ^16^ ^]^ Achieving an optimal receptor‐ligand binding affinity is critical to ensuring efficacy and specificity, as evidenced even in viral contexts.^[^ ^49^ ^]^ Instead of relying on affinity‐directed in vitro maturation to generate high‐affinity TCRs, we focus on enhancing the T cell reactivity of soluble 1–2C TCR engager proteins through two complementary strategies.

First, enhancing the binding avidity of TCRs by engineering multi‐valent scFv TCRs. For soluble TCR proteins, constructing multi‐valent TCRs would augment their binding avidity, potentially overcoming the limitations posed by low‐affinity TCRs. The extended lifetime of TCR engagement with pHLA could potentially facilitate the initiation of T cell activating signaling via the anti‐CD3 scFv antibody.

Second, T cell reactivity could be enhanced via site‐directed fine‐tuning of the TCR‐pMHC interaction.^[^ ^22^ ^]^ The αβ TCR functions as a mechanosensory, with TCR mediated T cell activation initiated by its engagement with pMHC.^[^ ^50^, ^51^ ^]^ During this process, TCR sensitivity could be precisely modulated through catch bond engineering, which maintained physiological affinities while enhancing T cell activation potency.^[^ ^22^ ^]^ Although the affinity increments of T96F‐mutated 1–2C TCR was minimal, maintaining physiological affinities characteristic of natural TCRs, the mutation significantly increased antigen sensitivity and T cell activation potency in T96F‐mutated 1–2C STanD‐scTCR engagers.

Moreover, the T96F‐mutated 1–2C STanD‐scTCR engager proteins demonstrated remarkable ability to induce significant higher frequencies of polyfunctional T cells, which are considered more competent in anti‐tumor immunity compared to monofunctional T cells.^[^ ^52^ ^]^ Additionally, the T96F‐mutated 1–2C STanD‐scTCR engager exhibited substantial tumor suppression in a tumor‐bearing mouse model. Therefore, the engineered T96F‐mutated 1–2C STanD‐scTCR engager represent a promising avenue for further development. However, the current study employed TCR engager proteins at relatively high concentrations, which may be attributed to the limited binding affinity of the incorporated TCR domains. The relative affinities between TCR and anti‐CD3 components critically influence the structural design of engager proteins. While enhancing TCR affinity could reduce the required clinical dosage, this optimization necessitates careful engineering to balance therapeutic potency with potential off‐target effects arising from affinity maturation. A limitation of TCR engager is HLA restriction that 1–2C TCR could only function in the context of HLA‐A*11:01. The KRAS‐G12V (VVGAVGVGK) can also be presented by other HLA‐A3 superfamily members with similar conformation. To broaden the therapeutic application of this TCR engager, future engineering of TCRs that could cross‐recognize KRAS‐G12V peptide presented by additional HLA alleles would be valuable. The two engineering strategies employed in this study, multi‐valency and site‐directed fine‐tuning, may serve as a foundation for designing next‐generation soluble TCR engagers, paving the way for the advancement of TCR‐based immunotherapeutics.

## 4 Experimental Section

### Cell Lines, T Cells and Peptides

K562 cells, PANC‐1 cells, CFPAC‐1 cells and SW‐620 cells were obtained from the Cell Resource Center, Institute of Basic Medical Sciences, PUMC (Beijing, China). SW‐620 cells and PANC‐1 cells were cultured in DMEM (Gibco) supplemented with 10% Fetal Bovine Serum (FBS) (Gibco). K562 cells and Jurkat cells (E6‐1 clone, ATCC, TIB‐152) were cultured in RPMI 1640 (Gibco) supplemented with 10% FBS (Gibco). CFPAC‐1 cells were cultured in IMDM supplemented with 10% FBS (Gibco). were cultured in RPMI 1640 (Gibco) supplemented with 10% FBS (Gibco). All the cells mentioned above were cultured in medium with 100 µg/mL streptomycin and 100 IU/mL penicillin (Invitrogen).

T cells were isolated from healthy donors and isolated with density centrifugation using lymphocyte Separation Medium (Dakewe Biotech). The use of the human T cells from healthy donors was reviewed and approved by the Institute of Microbiology, Chinese Academy of Sciences of Research Ethics Committee with assigned number authority APIMCAS2022076. Written informed consent was obtained from each of the donors. T cells were activated by adding 10 µL T Cell TransAct (Miltenyi Biotec) per 1 × 10^6^ cells and cultured in UniEx T‐cell serum‐free medium (ExCell) supplemented with 2.5% Helios UltraGRO‐Advanced (AventaCell), 300 IU mL^−1^ human IL‐2 (Novoprotein), 10 ng mL^−1^ human IL‐7 (Novoprotein), 100 µg mL^−1^ streptomycin and 100 IU mL^−1^ penicillin (Invitrogen). After 12 days of culture, the T cells were cryopreserved in FBS containing 10% dimethyl sulfoxide (DMSO) (Amiresco) at a concentration of 1×10^7^ cells per vial.

HEK‐293F cells were cultured with SMM 293‐TII Expression Medium (Sinobiological). All the cells were cultured at 37 °C in a 5% CO_2_ environment.

The peptides in this study were synthesized by the business (Genscript, Nanjing) and purified to 98% purity by high‐performance liquid chromatography (HPLC). Peptides were dissolved in DMSO (Amiresco) and diluted in cells medium before use.

### pHLA Tetramer Preparation

The preparation of HLA‐A*11:01‐restricted tetramers with KRAS‐G12V or KRAS‐G12wt peptide has been described in previous studies.^[^ ^53^ ^]^ Briefly, the extracellular domain of HLA‐A*11:01 (GenBank: AZL48402.1‐276) was modified by fusing a substrate sequence for the biotinylating enzyme BirA at the C‐terminus of the α3 domain. The modified HLA‐A*11:01 with the mutation of A245V and β2‐microglobulin were produced in *E. coli*. (BL21) as inclusion bodies. pHLA proteins were refolded in the refolding buffer consisting of 100 mm Tris‐HCl (pH 8.0) (Novon), 400 mm L‐arginine HCl (Amresco), 2 mm Ethylenediaminetetraacetic acid disodium salt (EDTA_2_Na) (Xilong Scientific), 10% glycerol (Concord), 5 mm reduced glutathione (GPC), 0.5 m oxidized glutathione (GPC) and 0.5 mm phenyl methane sulfonyl fluoride (PMSF) (Yuanye Bio) at 4 °C. The refolded pHLA proteins were purified by Superdex 200 Increase 10/300 GL column (Cytiva). The purified pHLA proteins were biotinylated for 12 h at 4 °C with D‐biotin, ATP, and the biotin protein ligase BirA (GeneCopoeia). The tetramers were assembly by incubating the biotinylated pMHC with PE‐streptavidin (BD Bioscience) at a molar ratio of 5:1 overnight at 4 °C. Tetramers were stored at 4 °C in PBS (10 mm Na_2_HPO_4_, 2 mm KH_2_PO_4_, pH 7.4, 137 mm NaCl, 2.7 mm KCl), 2 µg/mL Pepstatin (Sigma‐Aldrich), 2 µg mL^−1^ leupeptin (Sigma‐Aldrich), 4 mm EDTA_2_Na (Xilong Scientific) and 0.12% NaN_3_ (Sigma‐Aldrich).

### Generation of 1–2C TCR Engager Proteins

The design and constructs of 1–2C TCR engagers are detailed in Figure 1. These constructs were subjected to codon optimization for the mammalian cells expression and then synthetically integrated into the pCAGGS vector. The plasmids encoding the TCR engagers were transfected into HEK‐293F cells with polyetherimide (PEI) (Yesen). Transfected cells were cultured in SMM 293‐TII Expression Medium (Sinobiological) and supplemented with 35 mL SMS 293‐SUPI Expression Medium Supplement (SinoBiological) per liter at 24 and 72 h post‐transfection. On the fifth day, the supernatants were harvested by centrifugation and filtered through 0.22 µm membrane. The proteins were initially purified by affinity chromatography using protein A column (Cytiva) or HisTrap excel column (Cytiva), and further purified by gel‐filtration chromatography using Hiload 16/600 Superdex 200 pg (Cytiva) in PBS buffer.

### Flow cytometry

The following antibodies were used in this study: anti‐hCD3 (UCHT1, Biolegend), anti‐hCD8 (SK1, BioLegend), anti‐hIFN‐γ (B27, BioLegend), anti‐hIL‐2 (MQ1‐17H12, BioLegend), anti‐hTNF‐α (MAb11, BioLegend), anti‐His (AD1.1.10, R&D Systems) and Hochest 33 342 (100×, Beyotime). T cells bound to 100 nM 1–2C TCR engager proteins were stained with 0.05 µg of pHLA tetramer per 1 × 10^6^ cells or other fluorescently labeld antibodies diluted at a 1:100 ratio. Staining was performed for 30 min at room temperature in flow cytometry (FACS) buffer which consisted of PBS supplemented with 2% FBS. Samples were analyzed by FACS Aria II (BD Bioscience) or FACS Calibur flow cytometer (BD Bioscience). FlowJo software (version 10) was used to analyze the data.

### IFN‐γ ELISPOT Assay

T cell responses against KRAS‐G12V induced by 1–2C TCR engagers were detected by IFN‐γ‐specific enzyme‐linked immunospot assay (ELISPOT) (BD Bioscience) and enzyme‐linked immunosorbent assay (ELISA) (BD Bioscience) as previously described.^[^ ^54^ ^]^ In brief, 96‐well ELISPOT plates were coated overnight at 4 °C with anti‐human IFN‐γ antibody diluted in PBS. T cells (5 × 10^4^ cells per well) and TCR engager proteins were co‐cultured with K562‐HLA‐A11 cells (1×10^4^ cells per well) loaded with KRAS‐G12V or KRAS‐G12wt peptides in the wells for 18 h at 37 °C. T cells stimulated with PMA/ionomycin (Dakewe Biotech) served as a positive control for non‐specific stimulation, while T cells incubated with medium alone were used as a negative control. After incubation, cells were removed and the plates were processed according to the manufacturer's instructions. Spot‐forming units (SFUs) were counted using the ELISPOT readers analyzers software (Immunospot).

### IFN‐γ ELISA Assay

For antigen sensitivity and TCR engager sensitivity assays, T cells (5 × 10^4^ cells per well) and 1–2C TCR engager proteins (100 nM) were co‐cultured with K562‐HLA‐A11 cells (1 × 10^4^ cells per well) loaded with either serial dilutions of KRAS‐G12V or G12wt peptides for 24 h. Alternatively, dose‐response assay was performed using a fixed peptide concentration (10 µg mL^−1^) with serial dilution of TCR engager proteins. For assays involving the positional scanning combinatorial peptide library, homologous peptides and allo‐HLA tumor cell lines, T cells (5 × 10^4^ cells per well) and 1–2C TCR engager proteins (100 nM) were co‐cultured with tumor cells (1 × 10^4^ cells per well) loaded with KRAS‐G12V or G12wt peptides (10 µg mL^−1^) for 24 h. The supernatant collected after co‐incubation was tested for secreted IFN‐γ using a commercial ELISA kit (BD Bioscience) following the manufacturer's protocol.

### Nano Differential Scanning Fluorimetry (NanoDSF)

1–2C TCR engager proteins at a concentration of 1 mg mL^−1^ were subjected to a thermal gradient ranging from 25 to 95 °C using the Prometheus NT.48 nanoDSF (NanoTemper Technologies). Proteins containing aromatic amino acid residues (Trp, Tyr and Phe) show intrinsic fluorescence, which changes as the proteins undergo denaturation.^[^ ^55^ ^]^ The fluorescence intensity ratio (350/330 nm) was monitored, and the thermal denaturation process was evaluated by plotting the first derivative of this ratio. The thermal transition (unfolding) temperature (Tm) was determined from the peak of the derivative curve. Additionally, protein aggregation was assessed by measuring changes in scattering fluorescence intensity during the thermal scan. The first derivative of the scattering was plotted.

### TCR Engagers Stability Assay Under Acid‐Base Oxidation Stability Conditions

Stability of 1–2C‐LL, 1–2C‐2LL, and 1–2C‐KIH TCR engager proteins was evaluated under varying stress conditions. Acidic Condition: Proteins were incubated in 20 mM CH₃COONa (pH 3.5) for 4 h at 28 °C. Alkaline Condition: Proteins were incubated in 20 mM Tris (pH 9.0) for 48 h at 37 °C. Oxidative Condition: Proteins were incubated in a solution containing 20 mm ammonium acetate, 20 mM citric acid‐sodium citrate, and 0.5 mm hydrogen peroxide (H₂O₂) for 24 h at 37 °C. Following treatment, the proteins and untreated controls were diluted serially and incubated with Jurkat cells (2 × 10^5^ cells per sample). After washes twice, the cells were stained with anti‐hCD3 (UCHT1, BioLegend) and KRAS‐G12V/HLA‐A*11:01 pHLA tetramers. The samples were collected and analyzed by FACS Calibur flow cytometer (BD Bioscience). The EC_50_ of the treated and untreated TCR engager proteins for binding to the tetramer was determined to assess their stability under each condition.

### Bio‐Layer Interferometry (BLI)

Bio‐Layer Interferometry (BLI) assay was conducted using the Octet RED96 in buffer PBST (PBS+0.005% Tween 20). The biotinylated KRAS‐G12V/HLA‐A*11:01 pHLA was prepared similarly to pHLA tetramer production above, without PE‐streptavidin incubation. The biotinylated pHLA was immobilized to streptavidin (SA) biosensors (Sartorius). Serial two‐fold dilutions of 1–2C‐LL, 1–2C‐2LL and 1–2C‐KIH TCR engager proteins were prepared at concentrations ranging from 20 to 0.62 µm and added to 96‐wells plate designed for BLI analysis. The Octet RED96 instruments sequentially dipped the sensors into wells containing the varying concentrations of TCR engager proteins, and the binding were analyzed as changes in the biological layer thickness at the biosensor surface.

### Surface Plasmon Resonance (SPR)

SPR binding assays were conducted using a Biacore 3K instrument (GE Healthcare) with streptavidin (SA) chips (Cytiva). Six favorable residue mutations identified by Rosseta △G were introduced to the monomeric 1–2C‐LL TCR engager. These mutant 1–2C‐LL proteins were expressed and purified as described for 1–2C‐LL TCR engager proteins and buffer‐exchanged to PBST. Biotinylated KRAS‐G12V/HLA‐A*11:01 pHLA was immobilized on SA chip to a level of roughly 1000 response units (RU). Proportionate concentrations of mutant 1–2C‐LL engager proteins were flowed sequentially over the immobilized pHLA. BIAevaluation software (GE healthcare) was used for data analysis, and equilibrium dissociation constants (K_D_) values were calculated by fitting the data to a 1:1 binding model.

### Target Cells Preparation

K562, SW‐620, PANC‐1, CFPAC‐1 cells were genetically engineered and utilized as target cells to evaluate the specific T cell responses induced by 1–2C TCR engagers. K562 cells were engineered to express HLA‐A*11:01 by lentiviruses with HLA‐A*11:01 heavy chain and referred as K562‐HLA‐A11, serving as antigen‐presenting cells loaded with peptides. K562, SW‐620, PANC‐1, CFPAC‐1 cells were further engineered to over‐express HLA‐A*11:01 and KRAS‐G12V by lentiviruses with constructs encoding KRAS‐G12V‐β2m‐HLA‐A*11:01 heavy chain single‐chain trimer (SCT) and GFP. These engineered cell lines were designated as K562‐HLA‐A11‐KRAS‐G12V, SW‐620‐HLA‐A11‐KRAS‐G12V, PANC‐1‐HLA‐A11‐KRAS‐G12V, CFPAC‐1‐HLA‐A11‐KRAS‐G12V. Flow cytometry was used to assess the transduction efficiency by analyzing the frequency of GFP‐positive cells. The polyclone of GFP‐positive cells population was sorted and cultured. As SW‐620 intrinsically express KRAS‐G12V, they were also engineered to over‐express HLA‐A*11:01 through lentiviruses transduction with construct encoding HLA‐A*11:01 heavy chain and luciferase, and referred to as SW‐620‐HLA‐A11‐luci. Single‐clones of SW‐620‐HLA‐A11‐luci cells were sorted by flow cytometry and cultured for further application.

### Intracellular Cytokine Staining (ICS)

The ICS assay was conducted as previously described.^[^ ^54^ ^]^ Briefly, T cells (5 × 10^5^ cells per well) and 1–2C‐2LL TCR engager proteins (100 nM) incubated with K562‐HLA‐A11 cells (1 × 10^5^ cells per well) loaded with KRAS‐G12V or KRAS‐G12wt peptides (20 µg mL^−1^) in 96‐well plates for 6 h at 37 °C. T cells cultured with medium alone were enrolled as negative control while T cells stimulated with PMA/ionomycin (Dakewe Biotech) were adopted as positive control. All the wells were treated with GolgiStop (BD Bioscience) at the time of incubation to block cytokine secretion. After stimulation, the T cells were collected and stained with anti‐hCD8 (SK1, BioLegend) for 30 min at room temperature. Following surface staining, cells were fixed and permeabilized with BD Fix/Perm buffer (BD Science). Intracellular cytokines were then labeled with anti‐hIFN‐γ (B27, BioLegend), anti‐hTNF‐α (MAb11, BioLegend) and anti‐hIL‐2 (MQ1‐17H12, BioLegend). Following washes and re‐suspension, samples were examined with FACS Arial II and data were analyzed with FlowJo software (version 10).

### Flow Cytometry‐Based Cytotoxicity Assay

T cells (5 × 10^5^ cells per well) were incubated with 1–2C‐2LL TCR engager proteins (100 nM) and genetically engineered tumor cells (K562, SW620, CFPAC‐1, PANC‐1) over‐expressing HLA‐A*11:01 and KRAS‐G12V (1 × 10^5^ cells per well) in 96‐well plates for 48 h at 37 °C. T cells and tumor cells incubated with PBS served as negative control. After co‐culturing, cells were collected and stained with BD Horizon Fixable Viability Stain 780 (BD Bioscience) for 15 min at 4 °C to assess cell viability. The cells were washed and further stained with anti‐hCD3 (UCHT1, BioLegend) and anti‐hCD8 (SK1, BioLegend). Samples were washed, resuspended in FACS buffer, and CountBright absolute counting beads (10 µL per sample) (Thermo Fisher) were added for absolute quantification. Samples were analyzed using FACS Arial II flow cytometer, ensuring the collection of 500 counting beads per sample to ensure total cells consistently in quantity. The CD3^−^CD8^−^ cell population was identified and quantified as tumor cells. Data were analyzed with FlowJo software (version 10).

### Crystal Screening, Data Collection and Structural Determination

Crystal screening of 1–2C‐T96F TCR and KRAS‐G12V/HLA‐A*11:01 complex was set up with TCR and pHLA proteins expressed in *E. coli* cells.^[^ ^56^ ^]^ TCR protein expression and purification were carried out as previously described. Soluble 1–2C‐T96F TCR were obtained by a previously established in vitro refolding protocol. The refolded TCR proteins were purified via ion‐chromatograph using Source 15Q PE 4.6/10 column (Cytiva), followed by gel‐filtration chromatography with Superdex 200 Increase 10/300 GL column (Cytiva). The KRAS‐G12V/HLA‐A*11:01 proteins expression and purification were similar to the pHLA tetramer production without biotination. The 1–2C‐T96F TCR proteins were incubated with KRAS‐G12V/HLA‐A*11:01 at a molar ratio of 1:1.5 and the resulting TCR/pMHC complexes were purified using gel‐filtration chromatography. 1 µL complex proteins (10 mg mL^−1^) were mixed with 1 µL reservoir solution (Molecular Dimensions) and incubated at 18 °C using sitting drop vapor diffusion technique for screening complex protein crystal. 1–2C‐T96F/KRAS‐G12V/HLA‐A*11:01 crystals grew in reservoir solution containing 0.1 m Magnesium chloride hexahydrate, 0.1 m Sodium HEPES (pH 7.0), and 15% w/v PEG 4000.

For crystals data collection, the crystals were picked up using the nylon loop and then soaked in the reservoir solution supplemented with 17% v/v glycerol for a few seconds.^[^ ^57^ ^]^ Then transferred the crystals to liquid nitrogen to freeze. The crystal diffraction data were obtained from Shanghai Synchrotron Radiation Facility BL02U and the datasets were processed by XDS program.^[^ ^58^ ^]^ The TCR/pMHC complex structure were determined by the molecular replacement method (phaser MR) using CCP4 suite with the search model (PDB: 8I5C).^[^ ^59^ ^]^ The structure was built with COOT software and refined for several rounds of the refinement with PHENIX. The stereochemical quality of the final model were assessed with Molprobity.^[^ ^60^, ^61^, ^62^ ^]^ Data collection and refinement statistics are described in Table S1 (Supporting Information). Coordinate for 1–2C‐T96F/KRAS‐G12V/HLA‐A*11:01 has been deposited in the PDB (http://www.rcsb.org/pdb) under accession number 9IKY. Structure analyses were conducted with the PyMOL program (https://pymol.org).

### Immunofluorescence Imaging

Immunofluorescence imaging assay was carried out as previously described.^[^ ^63^ ^]^ T cells and 1–2C‐2LL TCR engager proteins (1 µM) were incubated with K562‐HLA‐A11 cells pre‐loaded with KRAS‐G12V or KRAS‐G12wt peptides (10 ug mL^−1^) for 1 h at 37 °C. After incubation, cells were gently washed twice with FACS buffer and stained with anti‐hCD8‐PE (SK1, BioLegend), anti‐His‐APC (AD1.1.10, R&D Systems) and Hochest 33 342 (Beyotime) at room temperature for 15 min. Stained cells were washed twice with FACS buffer and fixed in 4% paraformaldehyde (Solarbio) for 12 h at 4 °C. Fixed cells were imaged using cytek Amnis FlowSight Imaging Flow Cytometer, and images were analyzed by IDEAS software. Cells were gated on coupled cell area and doublet bright‐field parameters. Synapse area was defined by valley mask based on nucleus staining. The percentage of CD8 molecules or TCR engagers in/out of synapse was calculated by the following formula: intensity in synapse / (total intensity – intensity in synapse) * 100. PE fluorescence represents the intensity of CD8 molecules and APC fluorescence represents the intensity of TCR engagers.

### Cell Binding Avidity Assay

The cell binding avidity assay was conducted to evaluate the strength of interactions between T cells and target cells using the z‐Movi Cell Avidity Analyzer (Lumicks). SW‐620‐HLA‐A11‐luci cells were attached on poly‐L‐lysine (Sigma‐Aldrich) coated microfluidic chips for 4 h at 37 °C. T cells from healthy donors were labeled with CellTrace far red (ThermoFisher Scientific). The microfluidic chip containing the target cells was placed in the z‐Movi Analyzer. Labeled T cells were mixed with T96F‐mutated 1–2C‐2LL TCR engager proteins (1 µg mL^−1^) and the mixture was injected into the chip bounding with SW‐620‐HLA‐A11‐luci cells pre‐coated for 15 min before testing. Acoustic force was ramped from 0 N to 1000 N, gradually detaching bound cells. T cells incubated with PBS instead of engager proteins served as control. Detachment events were monitored, and data were analyzed using Ocean software.

### In Vivo Anti‐Tumor Activity in a Xenograft Tumor Model

Eight‐week‐old female NCG mice (Gempharma Tech, Nanjing, Strain NO. T001475) were employed for xenograft tumor model and the mice were housed in specific pathogen‐free conditions. In this experiment, Tumor burden was measured using bioluminescence imaging (BLI). The mice were inoculated with SW620‐HLA‐A11‐luci cells expressing luciferase into the back off the right side of each mouse subcutaneously. Next day, the mice were injected intraperitoneally with 150 mg kg^−1^ of the luciferase substrate D‐luciferin (PerkinElmer), dissolved in PBS. After 15 min, mice were anesthetized using isoflurane, and BLI was performed using the Xenogen IVIS system (PerkinElmer). BLI signals were extracted by measuring the brightness through typical circular regions of interest (ROIs) using the Living Image software. The total flux was computed and was recorded as photons per second (p/s). Mice with relatively homogeneous total flux were selected and divided into four groups, PBS treated, three varied doses of T96F‐mutated 1–2C‐2LL TCR engager proteins (10, 1, and 0.1 mg kg^−1^) treated. At day 2, T cells (2 × 10^7^ cells per mouse) from two separated healthy donors were injected intravenously accompany with IL‐2 (1 × 10^5^ U per mouse) injection Subcutaneously. At the same time, different doses of engager proteins were injected intraperitoneally and injection for consecutive 6 days and the ninth day. The tumor burden of the mice was monitored by BLI weekly. The endpoint for the experiment was defined as when tumor flux intensity exceeded 2 × 10¹⁰ p s^−1^ in any group, at which point all mice were sacrificed. All animal experiments were approved by the Committee on the Ethics of Animal Experiments of the Institute of Microbiology, Chinese Academy of Science (IMCAS) with assigned number authority APIMCAS2022075 and conducted in compliance with the recommendations in the Guide for the Care and Use of Laboratory Animals of IMCAS Ethics Committee.

### Conjugation of cy5.5 to 1–2C‐2LL‐T96F

1–2C‐2LL‐T96F TCR engager proteins were labeled with cy5.5 NHS Ester (AAT Bioquest) at a 1:10 (protein:dye) molar ratio in 200 mM Na_3_PO_4_ (pH 9.0) and incubated overnight at 4 °C in the dark. Unincorporated Cy5.5 was removed using a 30 KDa MWCO (Thermo Fisher Scientific), with extensive buffer exchange (>100‐fold) into PBS. The labeled TCR engager solution was sterilized using a 0.22 µm Ultrafree centrifugal filter (Millipore). The protein concentration and labeling efficiency were determined by Nanodrop fluorophore‐to‐protein (F/P) analysis, with final F/P ratios ranging from 1 to 2. The Cy5.5‐1‐2C‐2LL‐T96F was aliquoted and stored at −80 °C.

### Pharmacokinetic Analysis

Ten‐week‐old female NCG mice were administered Cy5.5‐1‐2C‐2LL‐T96F (10 mg kg^−1^) via either i.v. injection or i.p. injection. Blood was collected from treated mice (n = 2 or 3 per group) after 1/4, 3/4, 7/4, 6, 10, 12, and 24 h post‐injection. At each time point (except 10 h), Near‐infrared (NIR) fluorescence imaging was performed to monitor whole‐body distribution. An additional group of untreated mice (n = 3) served as negative control for background correction. Blood was obtained via retro‐orbital bleeding using heparinized capillary tubes, and plasma fluorescence intensity was measured using an Infinite 200 PRO Micro‐plate Reader (TECAN). The concentration of 1–2C‐2LL‐T96F TCR engager protein in plasma was determined by interpolation from a standard curve generated with known concentrations of the labeled protein.

### Statistical Analyses

Data analysis was performed using GraphPad Prism 9.0 (GraphPad Software). Data are presented as mean ± standard deviation (SD) or standard error of the mean (SEM). Statistical comparisons between groups were conducted using unpaired Student's t‐test or one‐way analysis of variance (ANOVA). At least three independent experiments were performed. Spearman's rank correlation was used to assess associations between variables. *p* value <0.05 were considered statistically significant (*p < 0.05, **p < 0.01, ***p < 0.001, ****p < 0.0001).

## Conflict of Interest

The authors declare no conflict of interest.

## Author Contributions

K.M., J.W., and M.J. contributed equally to this manuscript. S.T. and G.F.G. initiated and coordinated the project and designed the experiments; K.M., S.T., and G.F.G. analyzed the data and supervised the project; K.M., J.W., and J.H. expressed and purified the proteins; K.M. and J.H. conducted the SPR and BLI; K.M., D.L., and F.L. conducted the ELISA, ELISpot, and flow cytometry; K.M., M.J., F.L., and J.W. conducted the cell binding avidity assay; K.M., M.J., J.W., and F.L. conducted the animal experiments; Y.C., C.S., S.T., K.M., and J.H. solved the structure and analyzed the data; J.W., D.L., Y.C., C.W.Z., H.T., and X.M. analyzed and discussed the data; S.T., K.M., and G.F.G. wrote the manuscript. All authors revised the manuscript.

## Supporting information



Supporting Information

## Data Availability

The data that support the findings of this study are available from the corresponding author upon reasonable request.
