# Supplementary material for: An Engineered Soluble Single‐Chain TCR Engager for KRAS‐G12V Specific Tumor Immunotherapy
Source: Adv Sci (Weinh). 2025 Jun 5;12(31):e00181. doi: 10.1002/advs.202500181 (PMC12376689; doi:10.1002/advs.202500181)
Supplement: Supplementary file 1 — Supporting Information [file ADVS-12-e00181-s001.docx]

Supporting Information

**An engineered soluble single-chain TCR engager for KRAS-G12V specific tumor immunotherapy**

*Keke Ma, Jie Wang, Min Jiang, Juanhua He, Fangyang Li, Dan Lu, Chao Su, Yan chai, Wenjing Jin, Yu Chen, Catherine W.H. Zhang, Xiaopeng Ma, Hui Tan^*^, George F. Gao^*^, Shuguang Tan^*^*

.

**
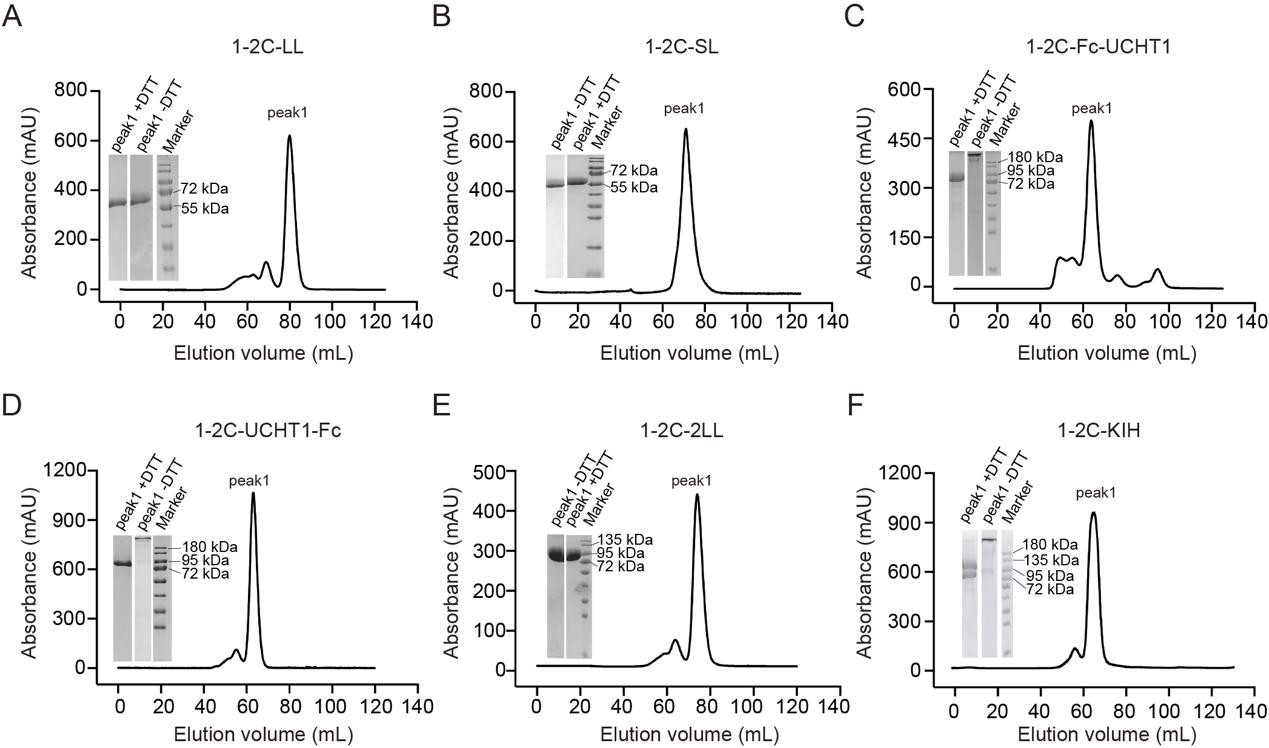
**

**Figure S1. Purification of the six soluble 1-2C TCR engager proteins.**

(A to F) Gel filtration profiles and SDS-PAGE analyses of 1-2C-LL (A), 1-2C-SL (B), 1-2C-Fc-UCHT1 (C), 1-2C-UCHT1-Fc (D), 1-2C-2LL (E) and 1-2C-KIH (F). Size-exclusion chromatography was performed using HiLoad 16/600 Superdex 200 pg column. SDS-PAGE analysis was conducted under reducing (+DTT) and non-reduced (-DTT) conditions. As shown in Gel filtration profiles and SDS-PAGE results, 1-2C-LL and 1-2C-2LL are present as monomers. 1-2C-SL, 1-2C-Fc-UCHT1, 1-2C-UCHT1-Fc and 1-2C-KIH are present as dimers.

**
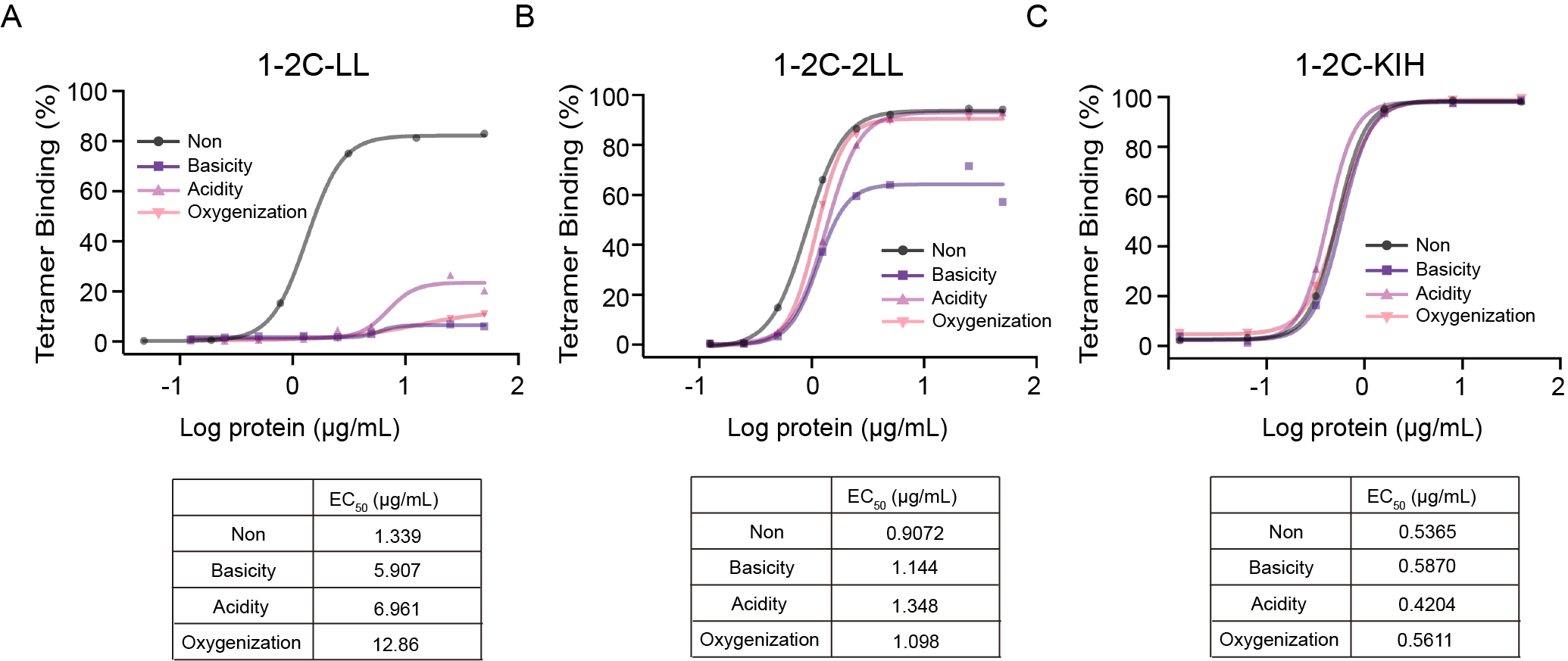
**

**Figure S2. Acid-base oxidation stability of 1-2C TCR engagers.**

(A-C) Stability of 1-2C-LL (A), 1-2C-2LL (B) and 1-2C-KIH (C) TCR engager proteins under acidic, alkaline and oxidative conditions over a defined period. Binding of pHLA tetramers loaded KRAS-G12V peptide to serial dilutions of 1-2C TCR engager proteins combination with Jurkat cells using flow cytometry. The EC_50_ values of 1-2C TCR engager proteins were determined to evaluate their binding affinity post-treatment.

**
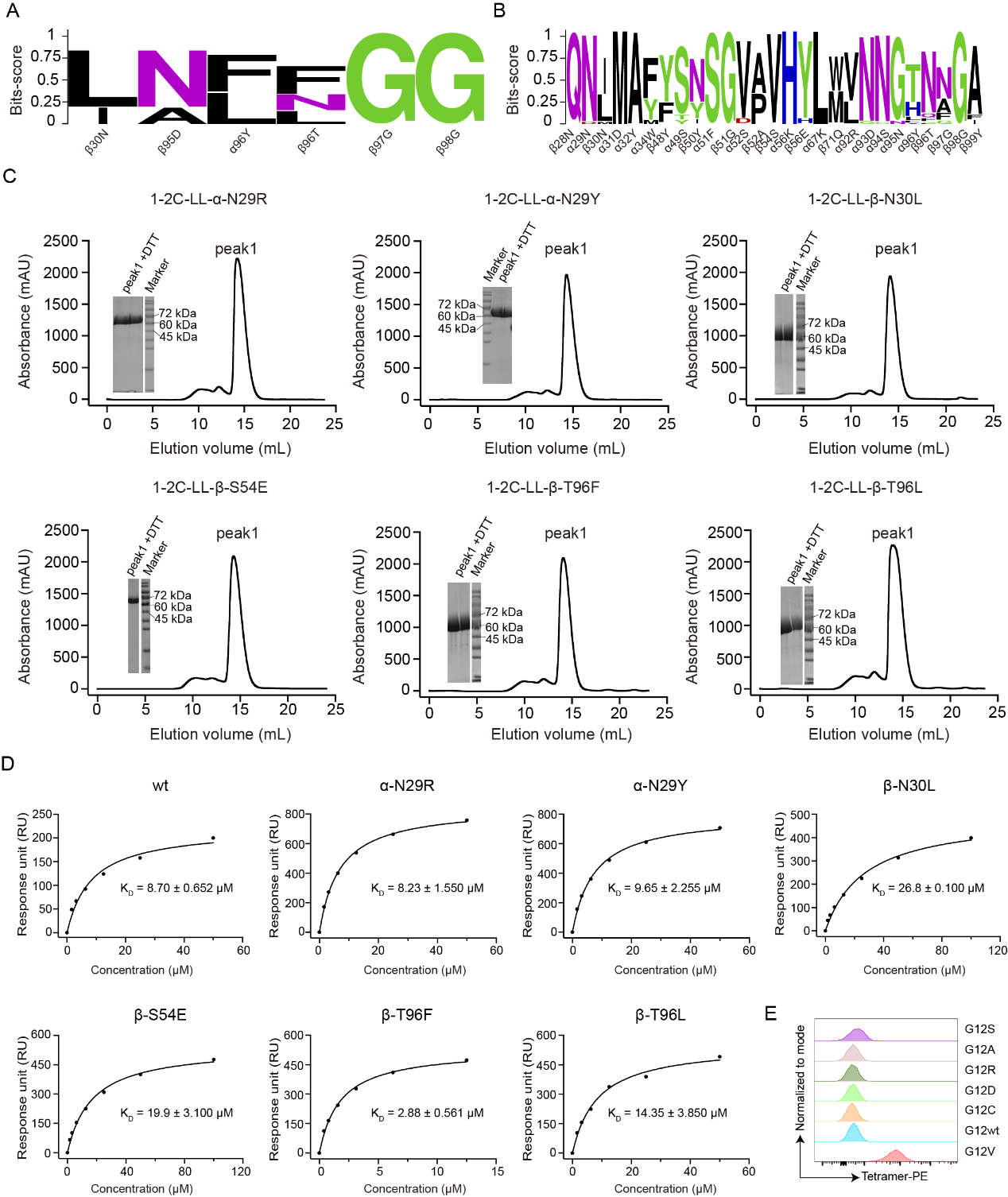
**

**Figure S3. Purification and binding analyses of six mutant 1-2C TCR proteins.**

(A-B) Sequence logos displaying potential amino acids designed to enhance the binding of the 1-2C TCR for the KRAS-G12V peptide (A) or for HLA-A*11:01 (B). Bits-score represented the frequency of specific amino acids at each site quantitatively. (C) Gel-filtration profiles of six 1-2C mutants integrated into 1-2C-LL construct analyzed by size-exclusion chromatography using Superdex 200 Increase 10/300 GL column. The corresponding SDS-PAGE analysis were performed under reducing (+DTT) conditions. (D) Equilibrium binding analysis of six site-mutant 1-2C proteins and wildtype (wt) 1-2C integrated into 1-2C-LL construct, with KRAS-G12V/HLA-A*11:01 pHLA. The results shown are representative of three independent experiments. (E) Binding of pHLA tetramers loaded with wildtype or varied KRAS-G12 mutant peptides to the T96F-mutated 1-2C-LL engager protein in combination with T cells, analyzed via flow cytometry. The data shown are representative of two independent experiments.

**
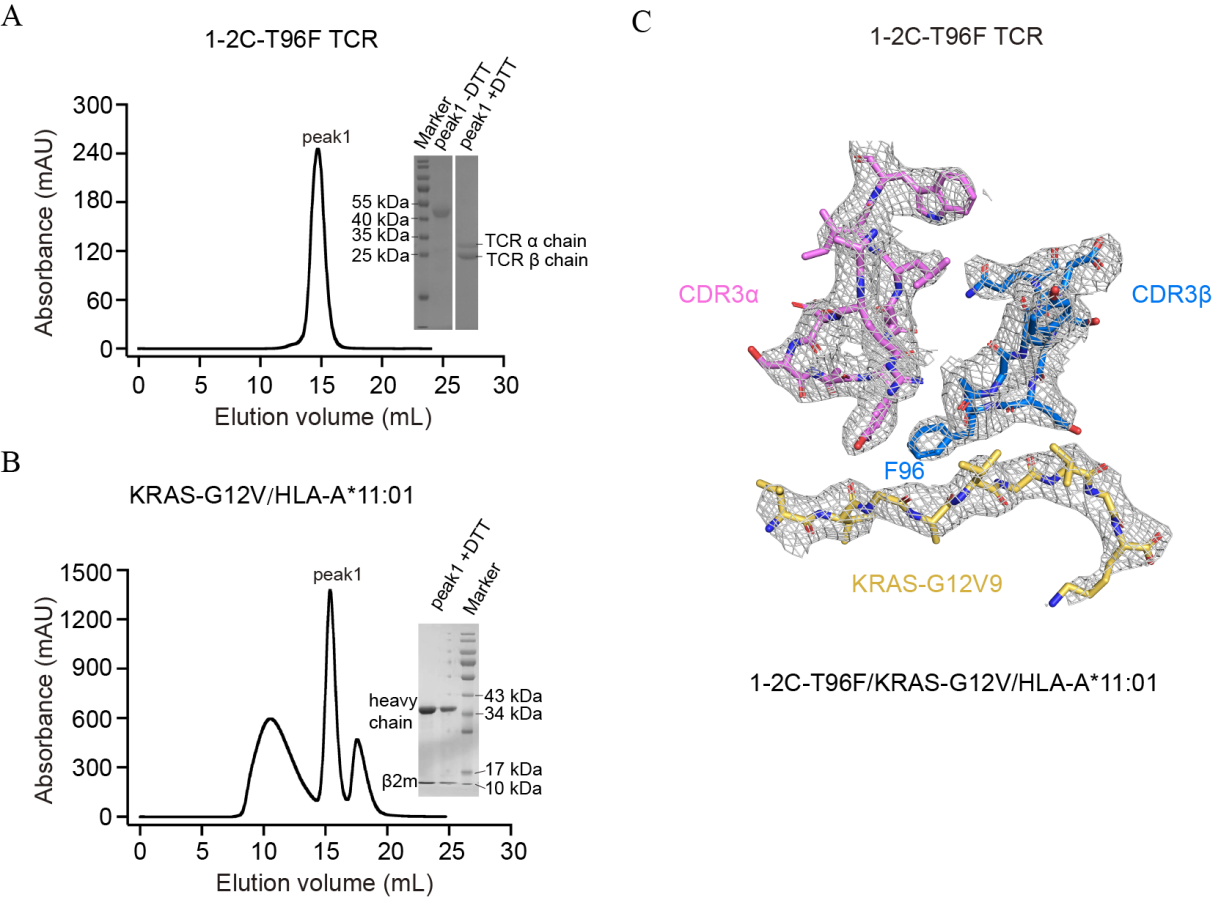
**

**Figure S4. Preparation the 1-2C-T96F TCR and** **KRAS-G12V/HLA-A*11:01 pHLA proteins, and electron density maps of binding interface in 1-2C/KRAS-G12V/HLA-A*11:01 complex.**

(A-B) Gel filtration profiles of 1-2C-T96F protein (A) and KRAS-G12V/HLA-A*11:01 (B) analyzed by size-exclusion chromatography using Superdex 200 Increase 10/300 GL. Corresponding SDS-PAGE analyses are shown.

(C) The electron density maps of binding interface between 1-2C-T96F TCR and KRAS-G12V in 1-2C-T96F/KRAS-G12V/HLA-A*11:01 complex. The density maps shown in grey 70 from the final 2Fo-Fc map are controled at 1σ. 1-2C-T96F CDR3α, violet; 1-2C-T96F CDR3β, marine; KRAS-G12V9, yellow.

**
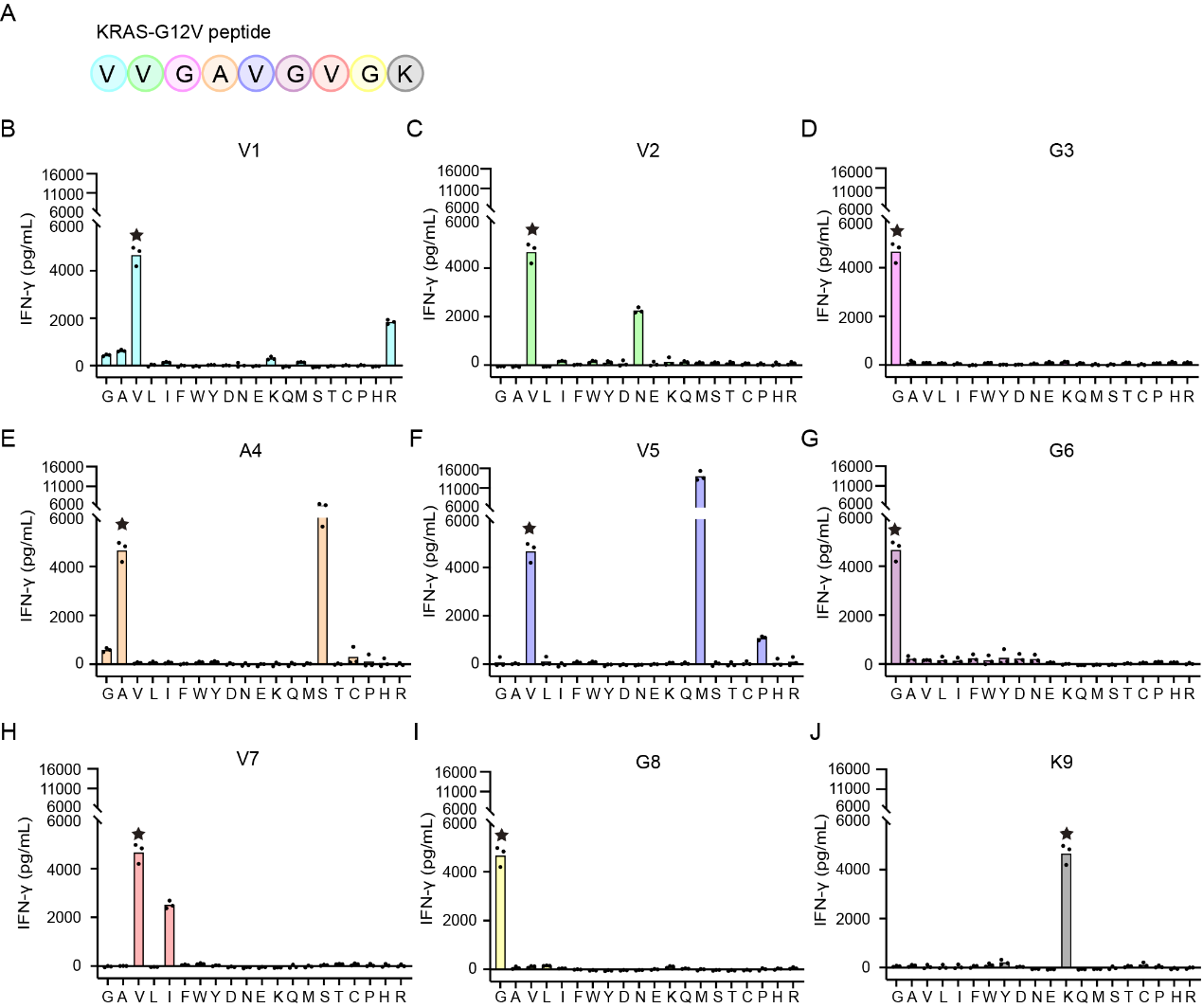
**

**Figure S5. Cross-responses of T96F-mutated 1-2C-2LL TCR engager to KRAS-G12V combinatorial peptide library.**

(A) Schematic representation of amino acids positions within the KRAS-G12V peptide. (B to J) Screening of 9-mer combinatorial peptide library to evaluate T cell responses induced by T96F-mutated 1-2C-2LL engagers specifically targeting the KRAS-G12V peptide. Co-cultures of T cells, T96F-mutated 1-2C-2LL engager proteins, and K562-HLA-A11 cells were tested for secreted IFN-γ responses in the presence of indicated peptides. The KRAS-G12V peptide is marked with a black star. IFN-γ levels were quantified using ELISA from triplicate supernatants for each condition. Dots represent three technical replicates from one representative donor out of two independently tested donors, and columns indicate the mean IFN-γ response.

**
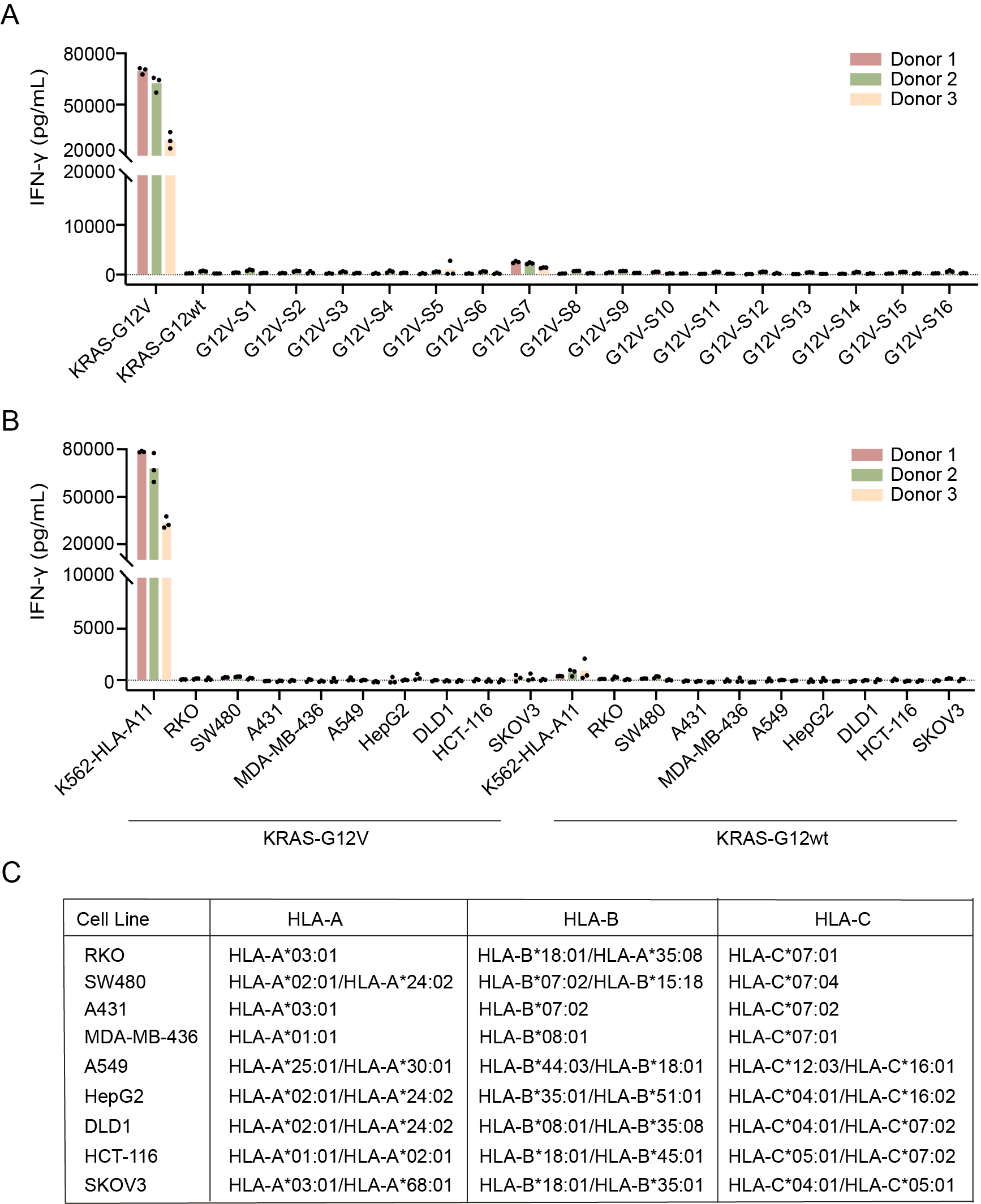
**

**Figure S6. Specificity of T****96F-mutated 1-2C-2LL TCR engager to homologous peptides in human genome and tumor cell lines expressing various HLA alleles**

(A) Recognition of homologous peptides by the T96F-mutated 1-2C-2LL TCR engager. KRAS-G12V and KRAS-G12wt peptides served as positive and negative control, respectively. The secreted IFN-γ were analyzed by ELISA assay with the co-culture supernatants of T cells, T96F-mutated 1-2C-2LL engager proteins, peptides, and K562-HLA-A11 cells. (B) Responses of the T96F-mutated 1-2C-2LL TCR engager against tumor cell lines with different HLA class I alleles, in the presence of KRAS-G12wt or KRAS-G12V peptide. IFN-γ secretion was measured by ELISA with the supernatants of co-cultures containing T cells, T96F-mutated 1-2C-2LL engager proteins, peptide, and tumor cell lines. In both (A) and (B), T cells were isolated from three individual donors (Donor 1–3). Each dot represents a technical replicate (n = 3 per donor, from a single experiment), columns indicate mean values. (C) HLA haplotypes for cell lines used in (B).

**
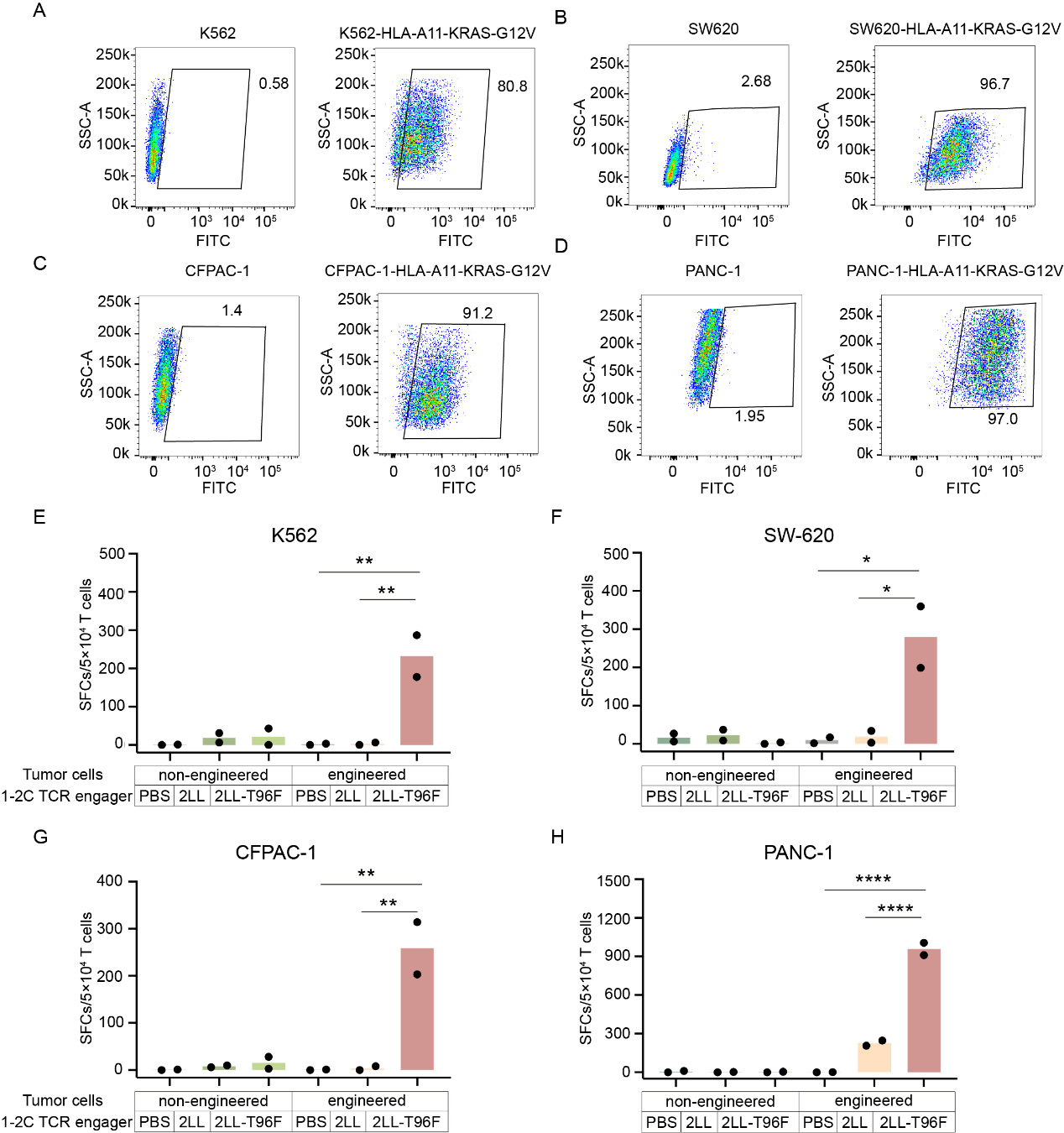
**

**Figure S7. Establishment and functional evaluation of target cell lines expressing KRAS-G12V/HLA-A*11:01.**

(A-D) Validation of expression efficiency of genetically constructed target cell lines. K562 cells (A), SW-620 cells (B), CFPAC-1 cells (C) and PANC1 cells (D) were transduced with KRAS-G12V-β2m-HLA-A*11:01, fused with GFP in the C-terminal, through lentiviral transduction. The modified cell lines, referred to as K562-HLA-A11-KRAS-G12V, SW-620-A11-KRAS-G12V, CFPAC-1-HLA-A11-KRAS-G12V, PANC1-HLA-A11-KRAS-G12V, were analyzed for GFP expression as a marker. (E-H). Evaluation of T cell immune responses against genetically modified tumor cells. T cells were co-cultured with 1-2C-2LL engager proteins (100 nM) or PBS (control) and tumor cells expressing KRAS-G12wt or KRAS-G12V. Responses were analyzed using IFN-γ ELISpot assays. Tumor cells without exogenous gene expression served as controls. Dots represent individual T cell responses from two separate donors, and columns indicate the mean number of IFN-γ-producing spots across donors. Statistical significance was determined using one-way ANOVA: ****P < 0.0001, **P < 0.01, *P < 0.05.

**
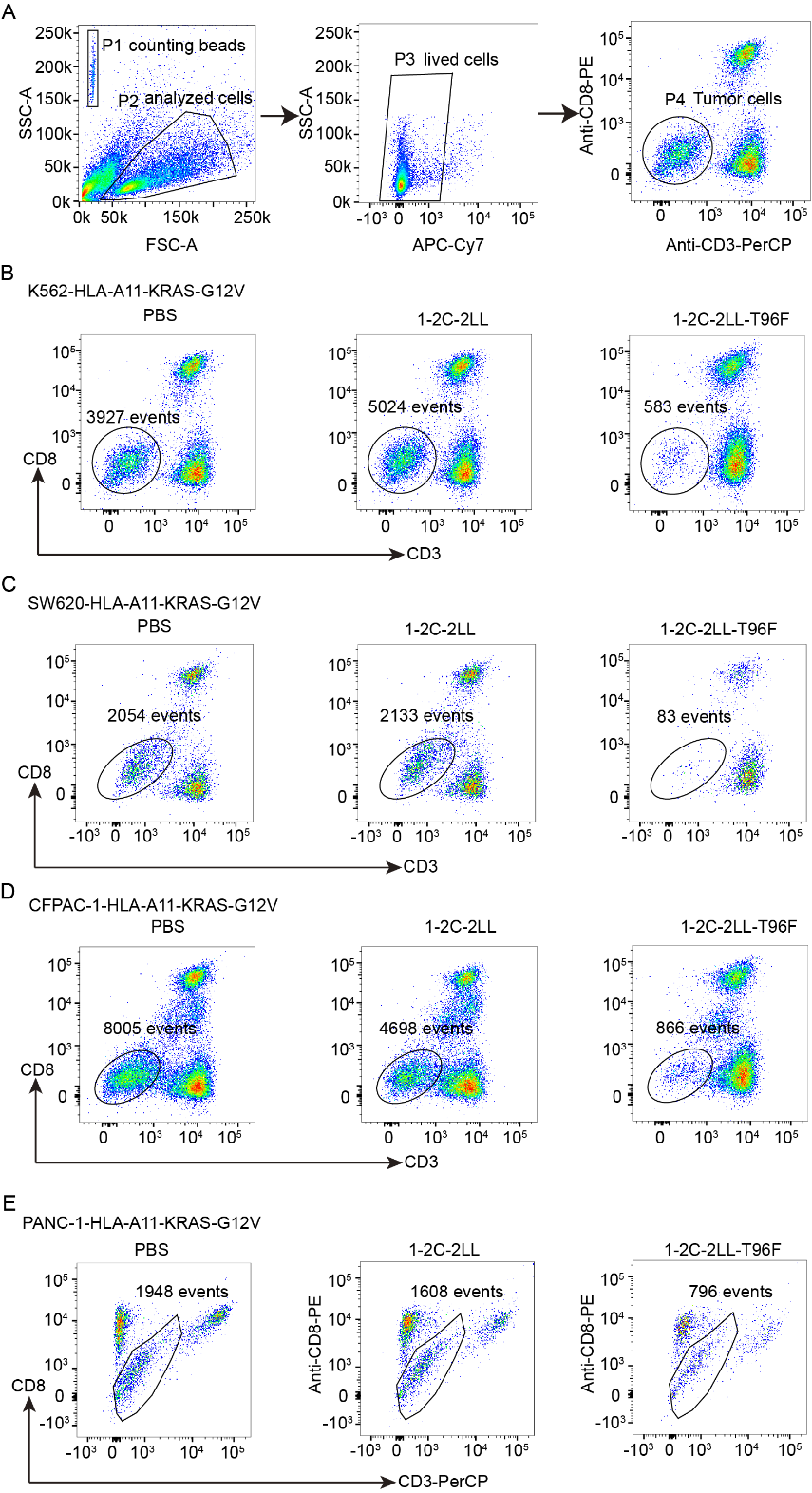
**

**Figure S8. Flow cytometry-based tumor cell killing assay.**

(A) Gating strategy for quantifying tumor cells in the flow cytometry-based killing assay. T cells and 1-2C-2LL TCR engager proteins were co-cultured with tumor cells engineered to express HLA-A*11:01 and KRAS-G12V. Counting beads (500 beads) were collected to quantify total T cells and tumor cells. Live cells were selected based on viability staining analysis. Tumor cells were identified as CD3^-^CD8^-^ populations. (B-E) Representative flow cytometry plots showing tumor cell killing by T cells co-cultured with 1-2C-2LL TCR engager proteins for K562-HLA-A11-KRAS-G12V (B), SW-620-A11-KRAS-G12V (C), CFPAC-1-HLA-A11-KRAS-G12V (D), PANC1-HLA-A11-KRAS-G12V (E). The inset numbers indicated the display event counts for the tumor cell population.


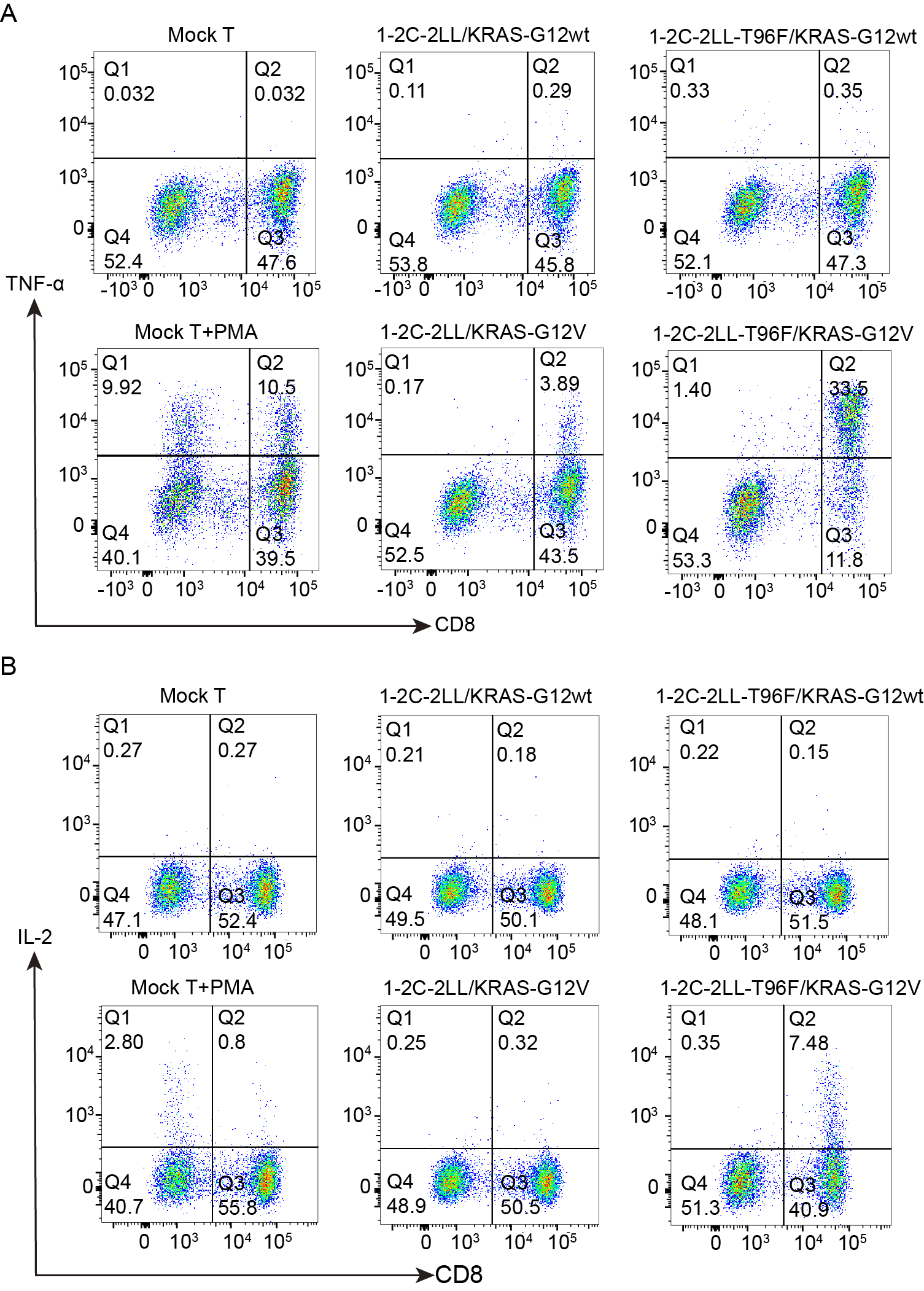


**Figure S9. Cytokine-producing profiles induced by 1-2C-2LL TCR engagers.**

Cytokine production by T cells following stimulation with wild-type or T96F-mutated 1-2C-2LL engagers in the presence of K562-HLA-A11 cells pulsed with KRAS-G12wt or KRAS-G12V peptides. Intracellular cytokine staining was performed for TNF-α (A) and IL-2 (B). The number in the panel represents the percentage of cytokine-staining positive cells.

**
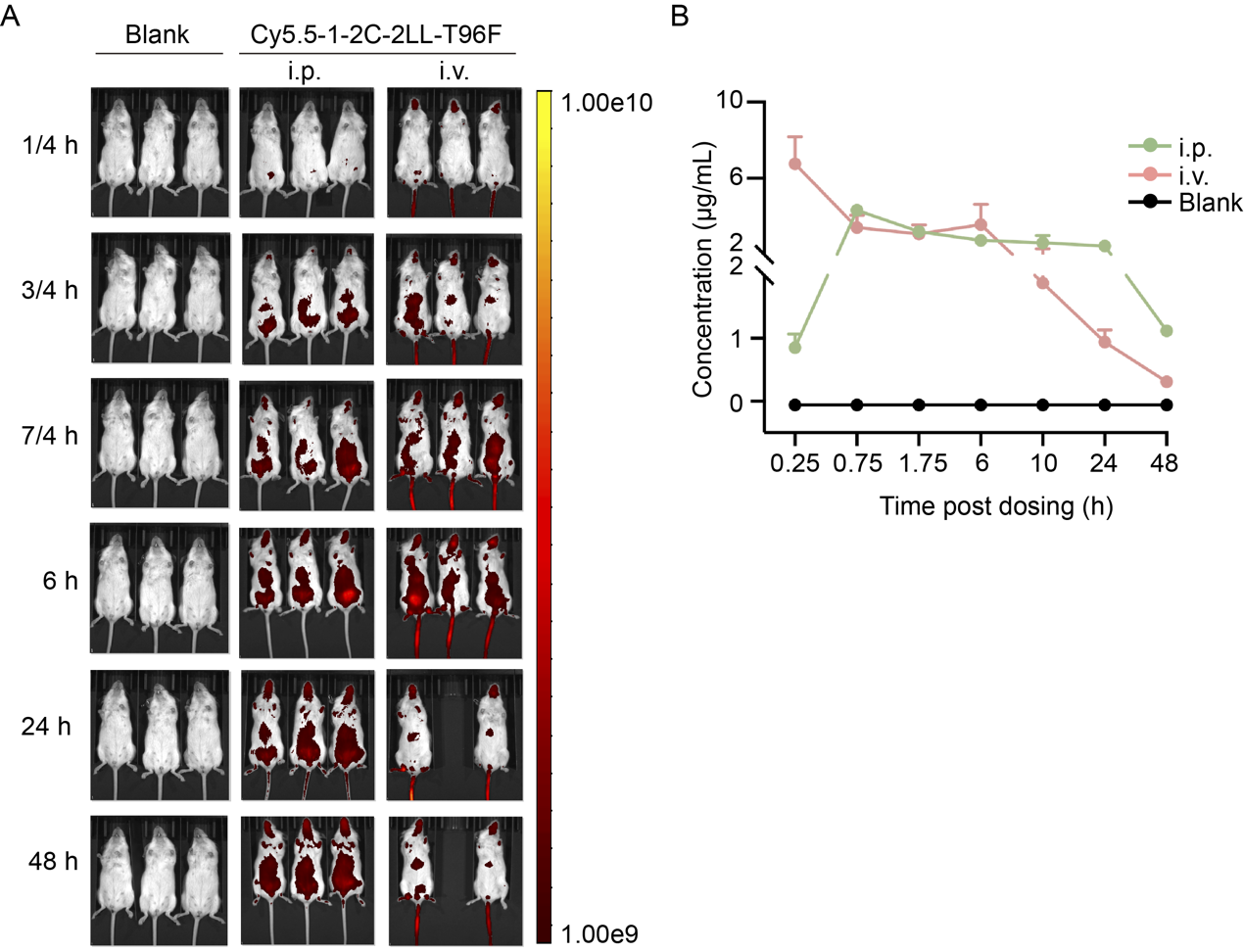
**

**Figure S10.** **Pharmacokinetic analysis of T96F-mutated** **1-2C-2LL TCR engager *in vivo*.**

(A) Whole-body near-infrared fluorescence images of mice at indicated time points following intraperitoneal (i.p.) or intravenous (i.v.) administration of Cy5.5-labeled 1-2C-2LL-T96F. Uninjected mice served as negative controls. (B) Quantification of T96F-mutated 1-2C-2LL TCR engager in plasma over time after i.p. or i.v. injection. Protein concentrations in plasma were determined based on a standard fluorescence calibration curve. Data represent mean ± SEM from at least two mice per group.

**
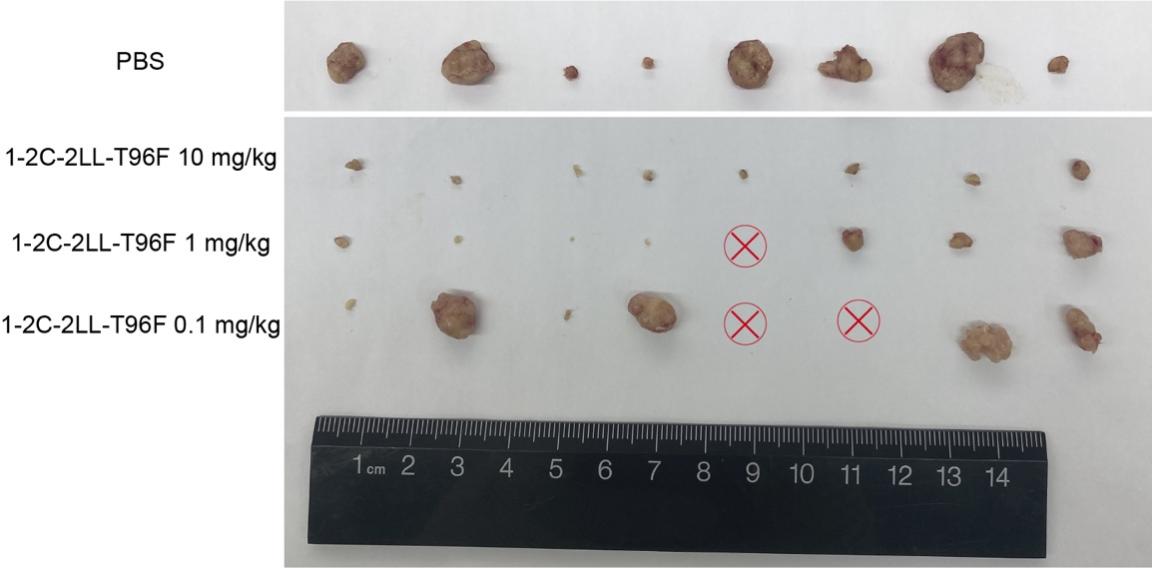
**

**Figure S11. Images of dissected tumors from mice after treatment.**

Dissected tumors from each mouse after euthanasia are shown for each indicated treatment group (n = 8 mice per group). A red “×” within a red circle denotes a mouse that did not survive until the end of the experiment.

**Table S1. Statistics for crystallographic data collection and structure refinement**

|  | 1-2C-T96F/ KRAS-G12V/HLA-A*11:01- |
| --- | --- |
| **Data collection statistics** |  |
| Space group | P1 |
| Cell dimensions |  |
| a, b, c (Å) | 157.775, 157.773, 193.023 |
| α, β, γ(°) | 103.027, 101.924, 82.165 |
| Resolution range (Å)^a^ | 63.43 - 3.12 (3.2 - 3.12) |
| R_merge_(%)^b^ | 14.1 (164.7) |
| I/σ(I) | 7.6 (0.9) |
| Completeness (%)^c^ | 98.20 (98.3) |
| Total observations | 1084726 |
| Unique observations | 308558 |
| **Refinement statistics** |  |
| R_work_ (%)^d^ | 21.17 |
| R_free_ (%) | 23.56 |
| No. of atoms |  |
| Protein | 8145 |
| Water | NA |
| rmsd from ideal values |  |
| Bond lengths (Å) | 0.003 |
| Bond angles (°) | 0.55 |
| Average B-factor (Å^2^) | 106.24 |
| Ramachandran plot statistics^e^ |  |
| Favored (%) | 96.20 |
| Allowed (%) | 3.59 |
| Disallowed (%) | 0.21 |
| Rotamer outliers (%) | 0.37 |
| Clashscore | 5.75 |

^a^ Values in parentheses refer to statistics in the outermost resolution shell.

^b^ Rmerge = $\sum_{\mathrm{hkl}}\sum_{i} \left| I_{i}-\left\langle I \right\rangle\right|\sum_{\mathrm{hkl}}\sum_{i} I_{i}$, where Ii is the observed intensity and $\left\langle I \right\rangle$is the average intensity of multiple observations of symmetry related reﬂections.

^c^ Data completeness = (no. of independent reflections) / (total theoretical number).

^d^ R =  $\sum_{\mathrm{hkl}} \left\| F\_obs\left| -k \right|F_{\mathrm{cal}} \right\|/\sum_{\mathrm{hkl}}\left| F_{\mathrm{obs}} \right|,$where Rfree is calculated for a randomly chosen 5% of reﬂections, and Rwork is calculated for the remaining 95% of reﬂections used for structure reﬁnement.

^e^ Ramachandran plots were generated by using the PROCHECK program of the CCP4i suite.

**Table S2**

**Contacts of HLA-A*11:01 with 1-2C TCR or 1-2C-T96F TCR**

| 1-2C/KRAS-G12V/HLA-A*11:01 | | | | 1-2C-T96F/ KRAS-G12V/HLA-A*11:01 | | | |
| --- | --- | --- | --- | --- | --- | --- | --- |
| Region | 1-2C  TCR | HLA-  A*11:01 | Contacts  (H bonds） | Region | 1-2C-T96F TCR | HLA-  A*11:01 | Contacts  (H bonds） |
| CDR1α | N29 | R163 | 2^a^ | CDR1α | N29 | R163 | 3 |
|  | D31 | R163 | 14 (2)^b^ |  | V30 | R163 | 1 |
|  | Y32 | Q155 | 11 (1) |  | D31 | R163 | 21 (3) |
|  | W34 | Q155 | 1 |  | Y32 | E154 | 1 |
| CDR2α | F51 | E154 | 4 |  |  | Q155 | 11 (1) |
|  |  | R157 | 5 |  |  | A158 | 2 |
|  |  | A158 | 13 |  | W34 | Q155 | 1 |
|  | K56 | E154 | 1 | CDR2α | F51 | E154 | 7 |
| CDR3α | R92 | Q155 | 1 |  |  | R157 | 7 |
|  |  | A158 | 1 |  |  | A158 | 7 |
|  |  | R163 | 2 |  | S52 | R157 | 1 |
|  | D93 | R163 | 5 (1) | CDR3α | R92 | Q155 | 1 |
|  | S94 | Q62 | 4 (1) |  |  | A158 | 1 |
|  |  | R163 | 4 |  |  | R163 | 3 (1) |
|  | N95 | Q62 | 3 |  | D93 | R163 | 8 (2) |
|  |  | R65 | 9 (1) |  | S94 | Q62 | 6 |
|  |  | N66 | 3 (1) |  |  | N66 | 1 |
|  |  | R163 | 1 |  |  | R163 | 5 |
|  | Y96 | N66 | 5 |  | N95 | Q62 | 7 |
|  |  | Y159 | 2 |  |  | R65 | 11 (3) |
|  |  | R163 | 10 |  |  | N66 | 4 |
| CDR1β | N28 | K146 | 1 |  |  | R163 | 3 |
|  | N30 | T73 | 9 (1) |  | Y96 | N66 | 6 |
|  |  | V76 | 2 |  |  | Y159 | 3 |
| CDR2β | Y48 | R65 | 4 |  |  | R163 | 6 |
|  | Y50 | R65 | 8 | CDR1β | N30 | N72 | 1 |
|  |  | N66 | 1 |  |  | T73 | 3 |
|  |  | A69 | 12 |  |  | V76 | 4 |
|  |  | Q72 | 7 | CDR2β | Y48 | R65 | 2 |
|  | G51 | Q72 | 5 |  | Y50 | R65 | 5 |
|  | A52 | Q72 | 3 |  |  | N66 | 1 |
|  | S54 | Q72 | 3 |  |  | A69 | 1 |
|  | E56 | R65 | 1 |  |  | Q72 | 4 |
| FR3β | Q71 | V76 | 2 |  | G51 | Q72 | 5 |
|  | T96 | A69 | 2 |  | A52 | Q72 | 2 |
| CDR3β |  | T73 | 1 |  |  | R75 | 3 |
|  | G97 | Q155 | 3 |  | E56 | R56 | 4 |
|  | G98 | Q155 | 12 (1) |  | Q71 | V76 | 3 |
|  | Y99 | A150 | 2 |  | F96 | N66 | 9 |
|  |  | H151 | 23 |  |  | A69 | 5 |
|  |  | E154 | 4 |  | G97 | Q155 | 9 |
|  |  | Q155 | 14 |  | G98 | Q155 | 10 (2) |
|  | | | |  | Y99 | A150 | 2 |
|  |  |  |  |  |  | H151 | 18 |
|  |  |  |  |  |  | E154 | 3 |
|  |  |  |  |  |  | Q155 | 14 |
| Total contacts | | 224 (9) | | Total contacts | | 237 (12) | |

a. Numbers represent the number of atom-to-atom contacts between 1-2C/1-2C-T96F TCR and HLA-A*11:01 residues, which were analyzed by the Contact program in CCP4 suite (the distance cutoff is 4.5 Å).

b. Numbers in the parentheses represent the number of hydrogen bonds between 1-2C/1-2C-T96F TCR and HLA-A*11:01 residues which were analyzed by the Contact program in CCP4 suite (the distance cutoff is 3.5 Å).

**Table S3**

**Contacts of KRAS-G12V with 1-2C TCR or 1-2C-T96F TCR**

| 1-2C/ KRAS-G12V/HLA-A*11:01 | | | | 1-2C-T96F/ KRAS-G12V/HLA-A*11:01 | | | |
| --- | --- | --- | --- | --- | --- | --- | --- |
| Region | 1-2C  TCR | KRAS-G12V-9 | Contacts  (H bonds） | Region | 1-2C-T96F  TCR | KRAS-G12V-9 | Contacts  (H bonds） |
| CDR3α | Y96 | V2 | 5 ^a^ (1) ^b^ | CDR3α | T96 | V2 | 6 (1) |
|  |  | G3 | 3 |  |  | G3 | 5 |
| CDR1β | N30 | G6 | 1 | CDR3β | D95 | G6 | 11 |
| CDR3β | D95 | G6 | 8 |  |  | V7 | 10 |
|  |  | V7 | 10 |  |  | G8 | 1 |
|  | T96 | V5 | 1 |  | F96 | A4 | 2 |
|  |  | G6 | 1 |  |  | V5 | 2 |
|  | G97 | V5 | 5 |  |  | G6 | 4 |
|  | G97 | V5 | 1 |  | G97 | V5 | 4 |
|  |  |  |  |  | G98 | V5 | 1 |
| Total contacts | | 35 | | Total contacts | | 46 | |

a. Numbers represent the number of atom-to-atom contacts between 1-2C/1-2C-T96F TCR and HLA-A*11:01 residues, which were analyzed by the Contact program in CCP4 suite (the distance cutoff is 4.5 Å).

b. Numbers in the parentheses represent the number of hydrogen bonds between 1-2C/1-2C-T96F TCR and HLA-A*11:01 residues which were analyzed by the Contact program in CCP4 suite (the distance cutoff is 3.5 Å).

**Table S4. Homologous peptides in human genome used in this study**

| **Name** | **Sequence** | **Resource** | **NCBI Reference Sequence** |
| --- | --- | --- | --- |
| G12V-9 | VVGAVGVGK | GTPase KRas isoform b [Homo sapiens] | NP_004976.2 |
| G12V-S1 | VVGAGGVSK* | carnosine synthase 1 isoform 1 [*Homo sapiens*] | NP_001159694.1 |
| G12V-S2 | VVGPVGCGK | ATP-binding cassette sub-family C member 3 isoform 1 [*Homo sapiens*] | NP_003777.2 |
| G12V-S3 | VVGGGGVGK | ras-related protein R-Ras [*Homo sapiens*] | NP_006261.1 |
| G12V-S4 | IVGRTGAGK | ATP-binding cassette sub-family C member 3 isoform 1 [*Homo sapiens*] | NP_003777.2 |
| G12V-S5 | VVGDGGVGK | ras-related protein M-Ras isoform 1 precursor [*Homo sapiens*] | NP_001078518.1 |
| G12V-S6 | VVGAVDSGK | tigger transposable element-derived protein 6 [*Homo sapiens*] | NP_112215.1 |
| G12V-S7 | VLGAPGVGK | ras-like protein family member 10A [*Homo sapiens*] | NP_006468.1 |
| G12V-S8 | VVGQVGCGK | multidrug resistance-associated protein 1 [*Homo sapiens*] | NP_004987.2 |
| G12V-S9 | VIGDLGVGK | ras-related protein Rab-38 [*Homo sapiens*] | NP_071732.1 |
| G12V-S10 | IVGCTGSGK | dynein heavy chain 2, axonemal isoform 1 [*Homo sapiens*] | NP_065928.2 |
| G12V-S11 | VVGNVGFGK | protein TANC1 isoform 1 [*Homo sapiens*] | NP_203752.2 |
| G12V-S12 | VVGGLAVGK | H(+)/Cl(-) exchange transporter 7 isoform a [*Homo sapiens*] | NP_001278.1 |
| G12V-S13 | VVGNAGTGK | dynein heavy chain 11, axonemal [*Homo sapiens*] | NP_001264044.1 |
| G12V-S14 | VIGPVGSGK | ATP-binding cassette sub-family C member 2 [*Homo sapiens*] | NP_000383.2 |
| G12V-S15 | VVGRTGAGK | ATP-binding cassette sub-family C member 2 [*Homo sapiens*] | NP_000383.2 |
| G12V-S16 | VVGPPGTGK | RNA helicase aquarius [*Homo sapiens*] | NP_055506.1 |

*The variant residues were highlighted with an underline.
